# Shared Genetics and Causality Between Decaffeinated Coffee Consumption and Neuropsychiatric Diseases: A Large-Scale Genome-Wide Cross-Trait Analysis and Mendelian Randomization Analysis

**DOI:** 10.3389/fpsyt.2022.910432

**Published:** 2022-07-11

**Authors:** Bian Yin, Xinpei Wang, Tao Huang, Jinzhu Jia

**Affiliations:** ^1^Department of Biostatistics, School of Public Health, Peking University, Beijing, China; ^2^Department of Epidemiology & Biostatistics, School of Public Health, Peking University, Beijing, China; ^3^Center for Intelligent Public Health, Academy for Artificial Intelligence, Peking University, Beijing, China; ^4^Key Laboratory of Molecular Cardiovascular Sciences (Peking University), Ministry of Education, Beijing, China; ^5^Center for Statistical Science, Peking University, Beijing, China

**Keywords:** coffee consumption, neuropsychiatric diseases, shared genetics, genetic correlation, Mendelian randomization, causality

## Abstract

Coffee or caffeine consumption has been associated with neuropsychiatric disorders, implying a shared etiology. However, whether these associations reflect causality remains largely unknown. To understand the genetic structure of the association between decaffeinated coffee consumption (DCC) and neuropsychiatric traits, we examined the genetic correlation, causality, and shared genetic structure between DCC and neuropsychiatric traits using linkage disequilibrium score regression, bidirectional Mendelian randomization (MR), and genome-wide cross-trait meta-analysis in large GWAS Consortia for coffee consumption (*N* = 329,671) and 13 neuropsychiatric traits (sample size ranges from 36,052 to 500,199). We found strong positive genetic correlations between DCC and lifetime cannabis use (LCU; Rg = 0.48, *P* = 8.40 × 10^−19^), alcohol use disorder identification test (AUDIT) total score (AUDIT_T; Rg = 0.40, *P* = 4.63 × 10^−13^), AUDIT_C score (alcohol consumption component of the AUDIT; Rg = 0.40, *P* = 5.26 × 10^−11^), AUDIT_P score (dependence and hazardous-use component of the AUDIT; Rg = 0.28, *P* = 1.36 × 10^−05^), and strong negative genetic correlations between DCC and neuroticism (Rg = −0.15, *P* = 7.27 × 10^−05^), major depressed diseases (MDD; Rg = −0.15, *P* = 0.0010), and insomnia (Rg= −0.15, *P* = 0.0007). In the cross-trait meta-analysis, we identified 6, 5, 1, 1, 2, 31, and 27 shared loci between DCC and Insomnia, LCU, AUDIT_T, AUDIT_C, AUDIT_P, neuroticism, and MDD, respectively, which were mainly enriched in bone marrow, lymph node, cervix, uterine, lung, and thyroid gland tissues, T cell receptor signaling pathway, antigen receptor-mediated signaling pathway, and epigenetic pathways. A large of TWAS-significant associations were identified in tissues that are part of the nervous system, digestive system, and exo-/endocrine system. Our findings further indicated a causal influence of liability to DCC on LCU and low risk of MDD (odds ratio: 0.90, *P* = 9.06 × 10^−5^ and 1.27, *P* = 7.63 × 10^−4^ respectively). We also observed that AUDIT_T and AUDIT_C were causally related to DCC (odds ratio: 1.83 per 1-SD increase in AUDIT_T, *P* = 1.67 × 10^−05^, 1.80 per 1-SD increase in AUDIT_C, *P* = 5.09 × 10^−04^). Meanwhile, insomnia and MDD had a causal negative influence on DCC (OR: 0.91, 95% CI: 0.86–0.95, *P* = 1.51 × 10^−04^ for Insomnia; OR: 0.93, 95% CI: 0.89–0.99, *P* = 6.02 × 10^−04^ for MDD). These findings provided evidence for the shared genetic basis and causality between DCC and neuropsychiatric diseases, and advance our understanding of the shared genetic mechanisms underlying their associations, as well as assisting with making recommendations for clinical works or health education.

## Introduction

Coffee, one of the most widely consumed beverages in the world, has received considerable attention on health consequences. Many different physiologically active compounds including caffeine, polyphenols, niacin, and others (1) have been associated with neuropsychiatric diseases. For example, coffee consumption was related to various mental diseases such as MDD (2), Alzheimer's (3) and Parkinson's disease (4), attention deficit and hyperactivity disorder (ADHD) (5), bipolar disorder (BIP) (6), and schizophrenia (SCZ) (7). One hypothesis to account for these associations is a common genetic liability. Genetic studies could provide insight into specific biological processes underlying comorbidity and disease risk.

With the increased availability of summary data from large-scale genome-wide association studies (GWAS) and methods estimating genome-wide genetic correlation by using only GWAS summary statistics (8–10), we can estimate the common genetic basis for complex traits by using genomics resources. Further, coffee consumption and neuropsychiatric diseases are heritable traits, and twin studies estimated the heritability of coffee consumption to be ~50% (11, 12). For different neuropsychiatric diseases, heritability has been estimated at varying between 50 and 80% (13–17). Recently, A GWAS of caffeine consumption in coffee consumers detected one susceptibility loci rs13107325 (SLC39A8) (18), which was also significantly identified in the GWAS of mental diseases (19, 20), suggesting potential shared genetic components between caffeine coffee consumption and neuropsychiatric diseases. Nevertheless, coffee is more than caffeine, and many other substances were found in amounts much more than that of caffeine and were thought to be responsible for health conditions (21–23). Little is known about the genetic correlations and shared genetic loci between DCC and neuropsychiatric diseases. In addition, traditional observational studies could be influenced by reverse causation or environmental confounders, and genetics investigation could help bridge these gaps from traditional epidemiological studies (24).

Therefore, we aimed to investigate the genetic correlation, causality, and shared genetic structure between decaffeinated coffee consumption and neuropsychiatric traits including insomnia, neuroticism, Alzheimer's disease (AD), BIP, anorexia nervosa (AN), MDD, SCZ, AUDIT score, ADHD, LCU, and amyotrophic lateral sclerosis (ALS) by using linkage disequilibrium (LD) score regression, bidirectional Mendelian randomization (MR), and genome-wide cross-trait meta-analysis with GWAS summary statistics, to provide more knowledge about the shared molecular mechanism of them and detect the potential genetic liability or strong evidence for a causal relationship.

## Materials and Methods

### Study Design and Data Sources

**Figure 1:**
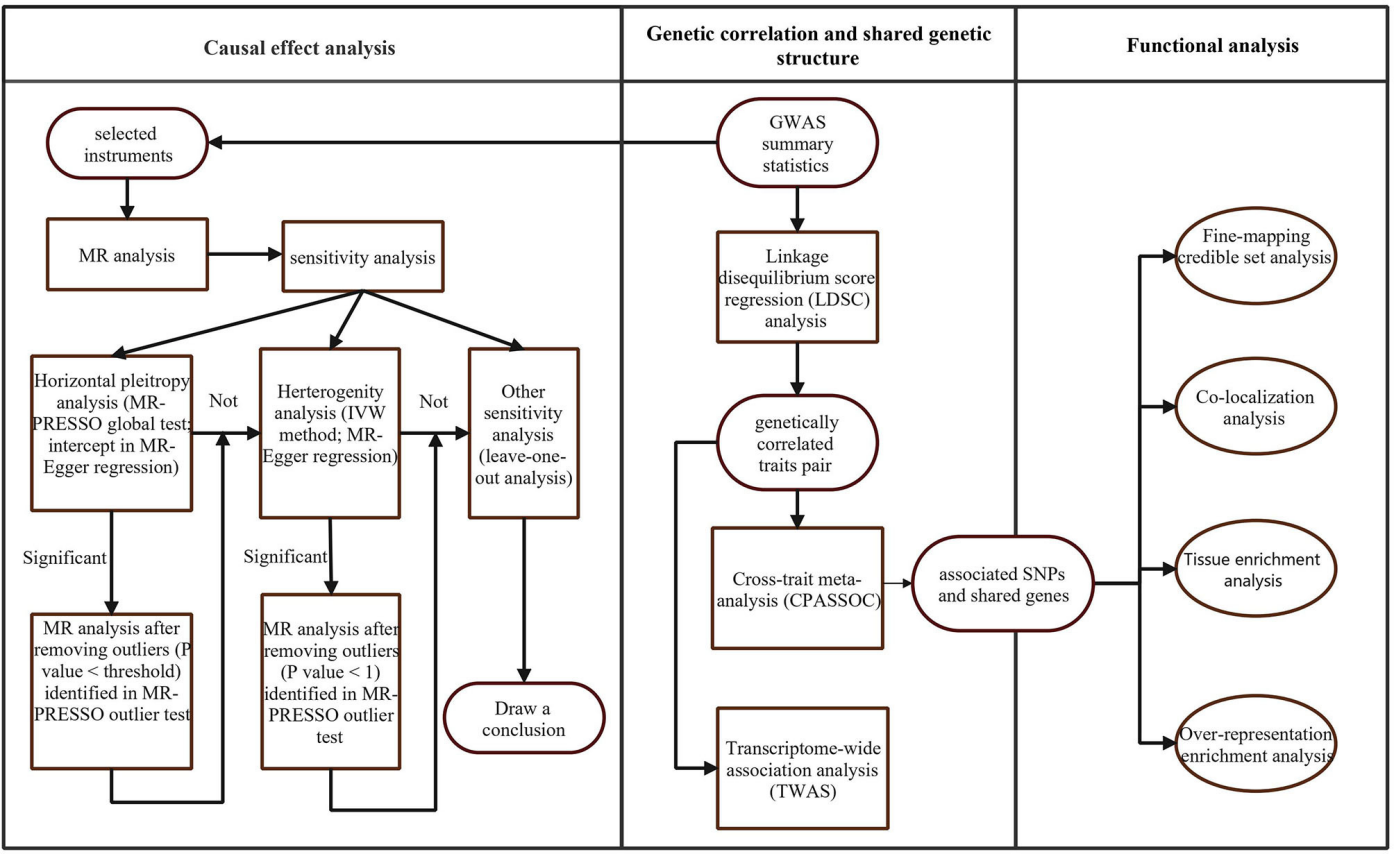
Overall study design.

The workflow of our analysis was shown in Figure 1. There were three main parts in our study: genetic correlation, causality, and shared genetics analysis between DCC and neuropsychiatric traits. We selected a large GWAS of coffee type in which the consumers of decaffeinated coffee were coded as cases, with the consumers of instant, ground, and other types of coffee coded as controls. Full summary statistics for coffee consumption (64,717 cases and 264,954 controls) were downloaded from the UK Biobank (UKBB) at http://www.nealelab.is/uk-biobank/. The GWAS summary statistics of insomnia and neuroticism items scores are available at https://ctg.cncr.nl/software/summary_statistics. We also could download GWAS summary statistics of AD (71,880 cases and 383,378 controls), BIP (20,352 cases and 31,358 controls), AN (16,992 cases and 55,525 controls), MDD (170,756 cases and 329,443 controls), SCZ (40,675 cases and 64,643 controls), and AUDIT (*n* = 121,604) from The Psychiatric Genomics Consortium (PGC) (Available online: http://www.med.unc.edu/pgc). Particularly, we selected three GWAS analysis results for AUDIT performed with AUDIT total score, AUDIT_C score, and AUDIT_P score. GWAS data on ADHD (19,099 cases and 34,194 controls) was derived from the meta-analyses by PGC and the Lundbeck Foundation Initiative for Integrative Psychiatric Research (iPSYCH) released in June 2017. The GWAS summary statistics for LCU (43,380 cases and 118,702 controls) and ALS (12,577 cases and 23,475 controls) were downloaded from the International Cannabis Consortium (ICC; Available online: https://www.ru.nl/bsi/research/group-pages/substance-use-addiction-food-saf/vm-saf/genetics/international-cannabis-consortium-icc/) and Project MinE, an international collaboration of investigators aiming to unravel the genetic basis of ALS, respectively (Available online: http://databrowser.projectmine.com/). For all the meta-analyses of GWAS included in this study, we only focused on the data generated from the analysis of individuals from Europe. The original GWAS studies can be referred to as the publications presented in Supplementary Table 1. A more detailed description of data has been described in their original studies; in addition, we restricted the chromosome region to autosomal chromosomes and excluded single-nucleotide polymorphisms (SNPs) in the MHC region (chromosome 6, base-pair positions 29,640,000–33,120,000 from the Genome Reference Consortium Human Build 37, hg19).

### Linkage Disequilibrium Score Regression Analysis

To evaluate the genetic correlation between DCC and neuropsychiatric traits, we conducted a post-GWAS genome-wide genetic correlation analysis by LD score regression using LDSC software (8, 9). The fact that the GWAS effect size estimate for each SNP represents the effects of all SNPs in LD with that SNP was met in this analysis. Thus, the SNPs in a high-LD region would show higher test statistics compared with the SNPs in a low-LD region. This method also provides a self-estimated intercept to show the overlapped subjects between studies.

### Cross-Trait Meta-Analysis

To identify shared genetic loci between traits pair that showed significant genetic correlations in LD score regression analysis, we performed a genome-wide cross-trait meta-analysis using CPASSOC (25). This method uses GWAS summary statistics of multiple traits with fewer limits of characteristics, either correlated, dependent, continuous, or binary traits coming from the same or different studies. Trait heterogeneity effects, population structure, and cryptic relatedness are also allowed. This method calculates two statistics, S_Het_ and S_Hom_. We used S_Het_ as the main statistic in consideration of the heterogeneity of different traits in our study. SNPs with *P*_SHet_ < 5 × 10^−8^ and trait-specific *P* < 0.01 were thought to have effects on both traits.

### Fine-Mapping Credible Set Analysis

To make the identified regions more precise, we conducted the fine-mapping credible set analysis. We extracted variants within 500 kb of the index SNP and identified a 99% credible set of causal variants at each of the shared loci between DCC and neuropsychiatric traits using the Bayesian likelihood fine-mapping algorithm (26). This method only maps the primary signal and uses flat prior with the steepest descent approximation. More details of the method were described in previous publications (27, 28).

### Co-localization Analysis

We used R “coloc” package to perform genetic colocalization analysis to detect whether the two traits we are interested in share common genetic variants in a given region. For each locus, we extracted variants within 500 kb of the index SNP. The probability H4 was calculated in this method and the co-localized loci were defined with a probability >0.5 (29–33).

### Tissue Enrichment Analysis

To calculate the tissue-specific gene enrichment and find out tissue of enriched expression of shared genes between DCC and neuropsychiatric traits, we performed a tissue enrichment analysis using the Tissue Enrich package (34, 35). The hypergeometric test was used in this analysis to determine if shared gene sets were highly enriched or specifically expressed in a tissue. The tissue-specific genes are enriched among the shared genes by processing RNA-Seq data from the Human Protein Atlas (HPA) and GTEx using the algorithm from the HPA.

### Over-representation Enrichment Analysis

We used the WebGestalt tool to assess the over-represented enrichment of the identified shared gene from a cross-trait meta-analysis in Reactome pathways and GO biological processes (36, 37). Benjamini–Hochberg procedure was used to account for multiple testing.

### Mendelian Randomization Analysis

We conducted a bidirectional MR analysis using a two-sample MR package to estimate if DCC and neuropsychiatric traits were causally related to each other, using inverse-variance weighted (IVW) (38) as the primary method. Other approaches including the simple median method, weighted median method, and MR-Egger method were also used to calculate the causal effect. The genetic instruments used in MR analysis must be associated with exposure and conditionally independent of the outcome given by the exposure, as well as not be the confounders between the exposure and outcome (24). To meet these consumptions, we chose SNPs with *P* < 1 × 10^−5^ from the GWAS summary statistics for interested exposure as the genetic instruments, and selected independent genetic instruments (clumping process *r*^2^ = 0.001, kb = 10,000). Considering the potential horizontal pleiotropy and heterogeneity, we used the intercept term in MR Egger regression to test a significant horizontal pleiotropy and used the IVW method and MR-Egger regression to detect heterogeneity. MR-Pleiotropy Residual Sum and Outlier (MR-PRESSO) method was also used as sensitivity analysis. We performed the MR analysis step by step as follows (39). First, we conducted an MR analysis with all the selected instruments. If the MR-PRESSO analysis indicated a significant horizontal pleiotropy, we shall perform MR analysis again after removing outlier variants with a *P*-value less than the threshold in the MR-PRESSO outlier test. After the above two steps, if the heterogeneity was still significant, we would remove all the SNPs with a *P*-value <1 in the MR-PRESSO outlier test and perform the final MR analysis. At last, we used the leave-one-out sensitivity analysis to identify potential influential SNPs and draw a robust and definitive conclusion or a conclusion with caution.

### Transcriptome-Wide Association Analysis

By using the FUSION software package based on 48 GTEx (version 7) tissue expression reference weights, we performed a TWAS to identify gene expression in specific tissues for each trait (40). The *P*-value was corrected by the false discovery rate (FDR) and Benjamin–Hochberg procedure, and FDR <0.05 was considered significant.

## Results

### Genome-Wide Genetic Correlation

We estimated the genetic correlation of DCC with 13 neuropsychiatric traits of interest. There were strong positive genetic correlations between DCC and LCU (Rg = 0.48, *P* = 8.40 × 10^−19^), AUDIT_T (Rg = 0.40, *P* = 4.63 × 10^−13^), AUDIT_C (Rg = 0.40, *P* = 5.26 × 10^−11^), AUDIT_P (Rg = 0.28, *P* = 1.36 × 10^−05^), and weak positive genetic correlation between DCC and SCZ (Rg = 0.10, *P* = 0.0117) (Table 1). In addition, DCC was negatively associated with neuroticism (Rg = −0.15, *P* = 7.27 × 10^−05^), MDD (Rg = −0.15, *P* = 0.0010), and insomnia (Rg = −0.15, *P* = 0.0007). No significant genetic correlations were observed of DCC with AD, ALS, ADHD, BIP, and AN (Table 1).

**Table 1:** Genetic correlation between decaffeinated coffee consumption and neuropsychiatric traits.

**Phenotype 1**	**Phenotype 2**	**Rg**	**Rg_SE**	***P*-Value**
Coffee consumption	Alzheimer's disease	−0.0033	0.0991	0.9732
	Amyotrophic lateral sclerosis	−0.0514	0.1294	0.6914
	Neuroticism*	−0.1533	0.0387	7.27E-05
	Major depression disorder*	−0.1469	0.0447	0.0010
	Insomnia*	−0.1523	0.0451	0.0007
	Attention deficit and hyperactivity disorder	−0.0579	0.0586	0.3228
	Bipolar disorder	0.0255	0.0444	0.5657
	Lifetime cannabis use*	0.4814	0.0544	8.40E-19
	AUDIT_T*	0.3926	0.0568	4.63E-13
	AUDIT_C*	0.3981	0.0606	5.26E-11
	AUDIT_P*	0.2769	0.0637	1.36E-05
	Anorexia nervosa	−0.0020	0.0637	0.9744
	Schizophrenia	0.1002	0.0397	0.0117

*Rg, genetic correlation estimate; SE, standard error; AUDIT_T, Alcohol use disorder identification test total score; AUDIT_C, alcohol consumption component of alcohol use disorder identification test item 1–3 scores; AUDIT_P, dependence and hazardous-use component of alcohol use disorder identification test item 4–10 scores*.

*Correlations that were significant after Bonferroni correction (P <0.05/13 traits = 0.0038).

### Cross-Trait Meta-Analysis

Considering the strong genetic correlation between DCC and seven neuropsychiatric traits, we performed a cross-trait analysis to improve our power to detect shared genetic loci between them. Shared genetic loci were defined by selecting SNPs with meta-analysis *P* < 5 × 10^−08^ and single-trait *P* < 0.05. In this analysis, we identified 73 independent loci totally, 32 of which were not genome-wide significant in single-trait GWAS of both DCC and neuropsychiatric traits (Table 2, Supplementary Table 2).

**Table 2:** Summary of the 73 genomic loci associated with decaffeinated coffee consumption and neuropsychiatric traits in cross-trait meta-analysis.

**Trait**	**Index SNP**	***N***	**CHR**	**POS**	**Kb**	**A1**	**A2**	**P1**	**P2**	***P*_meta_**
AUDIT_T	rs13107325	25	4	chr4:102702364..103387094	684.731	C	T	9.42E-17	2.94E-13	4.12E-23
AUDIT_C	rs13107325	24	4	chr4:102702364..103387094	684.731	C	T	9.42E-17	3.02E-10	6.87E-22
AUDIT_P	rs13107325	49	4	chr4:102702364..103387094	684.731	C	T	9.42E-17	7.87E-14	2.53E-24
	rs4371620*	1	4	chr4:102708235..102708235	0.001	C	T	3.87E-07	5.22E-03	4.58E-08
LCU	rs11214470*	1	11	chr11:112915628..112915628	0.001	A	G	1.69E-05	3.27E-05	3.15E-08
	rs12511373*	1	4	chr4:102666785..102666785	0.001	C	T	9.24E-07	2.74E-03	3.12E-08
	rs13107325	24	4	chr4:103112470..103374760	262.291	C	T	9.42E-17	1.63E-04	1.05E-19
	rs1368740	186	3	chr3:85403892..85792137	388.246	A	G	7.24E-05	1.34E-13	3.23E-13
	rs9919557	8	11	chr11:112827715..112915157	87.443	T	C	1.52E-03	6.53E-11	1.37E-10
MDD	rs10946917	65	6	chr6:27375611..27428991	53.381	C	T	2.42E-04	2.61E-10	6.42E-13
	rs1116690*	2	4	chr4:143500223..143510148	9.926	A	G	2.14E-03	4.46E-07	1.49E-08
	rs12438687*	2	15	chr15:96955776..96957969	2.194	C	T	3.86E-03	1.99E-07	9.20E-09
	rs12631196	133	3	chr3:157845169..158171455	326.287	A	G	9.10E-04	3.28E-08	4.86E-10
	rs1263674	30	2	chr2:208018534..208080975	62.442	T	C	4.48E-03	1.87E-08	9.86E-10
	rs12719532*	2	5	chr5:103795692..103822938	27.247	A	G	8.90E-03	8.11E-08	1.02E-08
	rs12967855	37	18	chr18:35127427..35251030	123.604	A	G	4.64E-03	1.50E-10	1.46E-11
	rs12969553	3	18	chr18:52873317..53101904	228.588	C	T	8.58E-03	8.20E-10	1.17E-10
	rs13107325	11	4	chr4:103112470..103292422	179.953	C	T	9.42E-17	3.88E-03	1.89E-16
	rs13353100*	6	13	chr13:53750354..53827237	76.884	G	T	8.57E-03	2.85E-07	2.65E-08
	rs150864553*	2	3	chr3:88072100..88136305	64.206	C	T	1.99E-05	1.45E-05	7.12E-09
	rs166418	7	9	chr9:17023857..17029942	6.086	C	T	7.42E-03	9.86E-09	1.14E-09
	rs2194028*	7	5	chr5:87775691..87865365	89.675	C	T	9.82E-06	6.01E-06	1.52E-09
	rs2276882*	5	4	chr4:3196029..3227592	31.564	T	A	2.37E-03	5.01E-07	1.18E-08
	rs4649957	51	1	chr1:72847620..72951344	103.725	A	T	6.08E-03	7.07E-13	1.96E-13
	rs4937872*	3	11	chr11:112827048..112912518	85.471	A	G	5.08E-04	1.28E-06	7.56E-09
	rs557042	214	6	chr6:27352750..27854760	502.011	C	T	5.42E-04	3.40E-17	5.76E-19
	rs56285315*	1	9	chr9:17421060..17421060	0.001	A	G	1.83E-04	6.49E-06	1.79E-08
	rs62401383	57	6	chr6:27318848..27769705	450.858	C	T	2.05E-04	2.81E-11	9.91E-14
	rs67777156	104	6	chr6:26354100..26824920	470.821	T	A	1.72E-03	4.76E-14	2.20E-15
	rs6783233	37	3	chr3:117484931..117709493	224.563	C	T	3.50E-03	7.07E-10	4.70E-11
	rs67981811	182	6	chr6:27854963..28411941	556.979	C	G	2.08E-03	1.14E-18	1.71E-19
	rs7147721	78	14	chr14:75123374..75377692	254.319	A	G	2.48E-04	2.79E-09	1.12E-11
	rs72676198*	65	1	chr1:67032354..67116043	83.69	C	A	5.63E-03	1.45E-07	9.28E-09
	rs72839477	58	6	chr6:26828359..27727513	899.155	C	T	2.40E-03	1.17E-15	8.71E-17
	rs7428847*	1	3	chr3:117529467..117529467	0.001	T	C	3.77E-03	1.09E-06	4.42E-08
	rs7525662	333	1	chr1:73284305..73722460	438.156	A	C	5.58E-03	5.32E-11	5.83E-12
Neuroticism	rs1050847	31	16	chr16:87396755..87444253	47.499	C	T	1.18E-03	1.15E-08	3.73E-10
	rs10759931*	2	9	chr9:120461621..120464147	2.527	G	A	5.20E-03	2.75E-07	3.94E-08
	rs10968658	53	9	chr9:28572233..28728183	155.951	A	C	5.25E-06	5.88E-09	4.04E-13
	rs11082011	75	18	chr18:35127427..35278126	150.7	C	T	4.66E-03	4.44E-21	7.16E-21
	rs12438687*	1	15	chr15:96955776..96955776	0.001	C	T	3.86E-03	1.66E-07	1.76E-08
	rs12969553	6	18	chr18:52972210..53101904	129.695	C	T	8.58E-03	1.43E-10	1.68E-10
	rs13107325	11	4	chr4:102702364..103292422	590.059	C	T	9.42E-17	6.51E-04	2.04E-16
	rs148696809*	2	6	chr6:28934352..29244219	309.868	T	C	1.86E-03	2.21E-07	9.19E-09
	rs1669025*	1	7	chr7:137069055..137069055	0.001	A	G	6.75E-03	6.37E-08	1.52E-08
	rs17671489*	3	18	chr18:35241254..35361130	119.877	G	C	2.87E-03	5.80E-07	3.59E-08
	rs17692129*	4	17	chr17:44793283..44848314	55.032	C	T	2.59E-03	5.39E-08	3.80E-09
	rs1892350	40	13	chr13:68021812..68121129	99.318	A	G	7.34E-03	7.00E-10	4.32E-10
	rs200477*	16	6	chr6:27656090..27769705	113.616	G	A	5.88E-05	1.54E-06	1.18E-09
	rs2067919*	12	5	chr5:87763468..87929869	166.402	T	C	1.24E-05	5.72E-08	9.25E-12
	rs2186710	135	11	chr11:112809652..112912811	103.16	C	G	5.09E-04	4.89E-12	1.84E-13
	rs28732378*	95	3	chr3:85403892..85784084	380.193	A	G	9.74E-06	1.69E-06	2.19E-10
	rs333453	20	1	chr1:174623770..174898715	274.946	T	A	9.20E-03	2.84E-09	2.00E-09
	rs34728579*	2	18	chr18:31515643..31610848	95.206	T	C	1.36E-03	9.45E-08	2.77E-09
	rs35641442	84	14	chr14:75070493..75384471	313.979	G	A	4.03E-04	1.10E-11	2.65E-13
	rs36092177*	12	6	chr6:28349698..28411941	62.244	C	T	2.09E-03	1.27E-07	6.08E-09
	rs3785235	6	16	chr16:7657673..7667392	9.72	A	G	7.06E-03	9.01E-12	1.29E-11
	rs3845980*	3	3	chr3:157515627..157901346	385.72	T	C	2.16E-03	7.62E-08	4.37E-09
	rs4679826*	1	3	chr3:158066761..158066761	0.001	G	A	5.13E-03	8.64E-08	1.36E-08
	rs483143	210	6	chr6:27357978..28345282	987.305	G	C	5.00E-04	3.73E-09	4.77E-11
	rs57713596	25	6	chr6:26903585..27327000	423.416	C	T	1.17E-03	4.49E-08	1.20E-09
	rs71566335*	28	6	chr6:100813469..100872703	59.235	A	G	1.52E-03	1.06E-07	3.83E-09
	rs7289932	5	22	chr22:41700361..41704872	4.512	A	G	8.23E-03	1.09E-08	4.67E-09
	rs73352630*	3	5	chr5:166061033..166065998	4.966	A	G	2.83E-03	9.47E-08	6.89E-09
	rs7772160	72	6	chr6:27318848..27736993	418.146	C	T	7.69E-04	2.88E-08	4.88E-10
	rs867328*	3	11	chr11:112913691..112915628	1.938	T	A	3.33E-06	3.18E-05	1.89E-09
	rs974711	1	4	chr4:90737327..90737327	0.001	G	A	9.37E-03	1.47E-08	7.70E-09
Insomnia	rs12969553*	1	18	chr18:53101904..53101904	0.001	C	T	8.58E-03	5.85E-08	1.19E-08
	rs13107325	11	4	chr4:102702364..103198082	495.719	C	T	9.42E-17	7.07E-03	2.11E-16
	rs2181389	66	13	chr13:53677723..53984714	306.992	A	G	4.85E-03	1.09E-10	2.73E-11
	rs55969935*	3	11	chr11:66646365..66650531	4.167	C	T	1.32E-05	4.06E-05	1.20E-08
	rs60751869	1	2	chr2:66816983..66816983	0.001	G	T	6.19E-04	2.96E-08	4.67E-10
	rs9576155	5	13	chr13:37527667..37620397	92.731	G	A	3.44E-03	9.26E-09	2.23E-09

*A1, alternative allele; A2, reference allele; P1, decaffeinated coffee consumption P; P2, neuropsychiatric traits P; CHR, chromosome; POS, position of the clumping region on the chromosome; SNP, single nucleotide polymorphism; MDD, major depression disorder; LCU, lifetime cannabis use; SCZ, Schizophrenia; AUDIT_T, Alcohol use disorder identification test total score; AUDIT_C, alcohol consumption component of alcohol use disorder identification test item 1–3 score; AUDIT_P, dependence and hazardous-use component of alcohol use disorder identification test item 4–10 score*.

^*^Not genome-wide significant in single-trait GWAS of both DCC and neuropsychiatric traits.

We identified two overlapped loci across all neuropsychiatric traits. One locus (index SNP: rs13107325) was identified in the genome-wide cross-trait meta-analysis of DCC with insomnia (*P*_meta_ = 2.11 × 10^−16^), neuroticism (*P*_meta_ = 2.04 × 10^−16^), LCU (*P*_meta_ = 1.05 × 10^−19^), AUDIT_T (*P*_meta_ = 4.12 × 10^−23^), AUDIT_C (*P*_meta_ = 6.87 × 10^−22^), AUDIT_P (*P*_meta_ = 2.53 × 10^−24^), and MDD (*P*_meta_ = 1.89 × 10^−16^). And rs13107325 was also the strongest signal observed for insomnia, LCU, AUDIT_T, AUDIT_C, and AUDIT_P. This locus was mapped to the protein-coding gene *SLC39A8*, which encodes for ZIP8, a divalent metal ion transporter. The encoded protein is glycosylated and found in the plasma membrane and mitochondria, and functions in the cellular import of zinc at the onset of inflammation (41, 42). The other locus (index SNP: rs12969553) was significantly associated with both DCC and insomnia (*P*_meta_ = 1.20 × 10^−08^), neuroticism (*P*_meta_ = 1.68 × 10^−10^), and MDD (*P*_meta_ = 1.17 × 10^−10^) in the cross-trait meta-analysis. This locus was close to gene *TCF4*, which encodes a helix-loop-helix transcription factor widely expressed throughout the body and during neural development and plays an important role in nervous system development. Moreover, polymorphisms in *TCF4* have been associated with many psychiatric and neurological conditions (43).

In addition to two overlapped loci, we identified 29 genome-wide significantly independent loci for DCC and neuroticism. The strongest association signal was observed on chromosome 18 at the *CELF4* region (index SNP rs11082011, *P*_meta_ = 7.16 × 10^−21^). *CELF4* was expressed primarily in excitatory neurons and encoded RNA-binding protein involved in pre- and postsynaptic neurotransmission, which played a vital role in human brain development and regulation of synaptic function (44, 45). The second strongest signal was the overlapped loci rs13107325. And the third strongest signal was mapped to *NCAM1* on chromosome 11 (index SNP rs2186710, *P*_meta_ = 1.84 × 10^−13^). This gene encoded a cell adhesion protein which is a member of the immunoglobulin superfamily. The encoded protein is involved in cell-to-cell interactions as well as cell-matrix interactions during development and differentiation and played a role in the development of the nervous system by regulating neurogenesis, neurite outgrowth, and cell migration, as well as in immune surveillance by the expansion of immune cells (46, 47).

In addition, we identified 27 genome-wide significant independent loci for DCC and MDD. The strongest association signal was mapped to zine finger protein genes (ZNFs), *OR2B6*, and *NKAPL* (index SNP rs67981811, *P*_meta_ = 1.71 × 10^−19^). Most of ZNFs encoded the proteins that are transcription factors that function by binding to specific DNA sequences and mediating protein-protein interactions. Zinc finger genes have been reported to be associated with psychiatric disorders (48). The protein encoded by *NKAPL* acted as a transcriptional repressor of Notch-mediated signaling required for T-cell development (49). *OR2B6* encoded Olfactory receptor proteins, which interact with odorant molecules in the nose, to initiate a neuronal response that triggers the perception of a smell. The second strongest signal was observed on chromosome 16, located near to histone family gene set (index SNP rs557042, *P*_meta_ = 5.76 × 10^−19^). Histones play a central role in transcription regulation, DNA repair, DNA replication, and chromosomal stability; these gene set also affects epigenetic pathways. The overlapped loci rs13107325 was the third strongest signal for DCC and MDD. The next strongest signal observed on Butyrophilin (BTN) family genes was another common finding in the genome-wide cross-trait meta-analysis of DCC and MDD (index SNP rs67777156, *P*_meta_ = 2.20 × 10^−15^). Among its related pathways are Butyrophilin (BTN) family interactions and Class I MHC-mediated antigen processing and presentation (20).

### Tissue Enrichment Analysis of Shared Genes

To identify the tissue of enriched expression of shared genes between DCC and neuropsychiatric traits, we performed a tissue-specific enrichment analysis using RNA-Seq data from the Human Protein Atlas (HPA) and GTEx. A total of 13 tissues were found with an enriched expression of shared genes between DCC and neuroticism, in addition to six tissues for MDD. The mainly enriched tissues contained bone marrow, lymph node, cervix, uterine, cerebellar cortex, lung, tonsil, and thyroid gland, and main part of the nervous system, exo-/endocrine system, and hemic and immune system. We also found nine and eight enriched tissues identified for shared genes of DCC and insomnia, and LCU, respectively. Tissues like the cervix, uterine, tonsil, appendix, lung, lymph node, and thyroid gland were enriched. Meanwhile, shared genes of DCC and AUDIT were enriched in two tissues, uterine cervix, and lung (Supplementary Figures 1–5).

### Over-representation Enrichment Analysis

The GO analysis showed that shared genes between DCC and neuroticism or MDD were significantly enriched in several shared biological processes, such as nucleosome organization, protein-DNA complex assembly, T cell receptor signaling pathway, and antigen receptor-mediated signaling pathway (Supplementary Table 4). Additional analysis of the Reactome pathway indicated that the association signals shared between DCC and neuroticism or MDD were significantly enriched in developmental biological, genetic, and epigenetic pathways, as well as immune-related pathways (Supplementary Table 5).

### Fine-Mapping and Co-localization Analysis

In fine-mapping analysis, we got lists of credible set SNPs shown in Supplementary Table 6 for each shared loci in the cross-trait meta-analysis results. The co-localization analysis showed that two loci (rs10968658, rs28732378) contained shared casual variant between DCC/neuroticism, in addition to two loci (rs13107325, rs1368740) for DCC/LCU, and one locus (rs150864553) for DCC/MDD. The loci for DCC/AUDIT all shared casual variants, which was consistent with the cross-trait meta-analysis (Supplementary Table 7).

### Mendelian Randomization (MR)

To examine whether there was evidence for the causal effect of DCC on neuropsychiatric disorders risk and vice versa, we performed a bidirectional two-sample Mendelian randomization analysis and got a robust MR analysis result shown after removing all the SNPs with a *P*-value <1 in the MR-PRESSO outlier test (Table 3, Supplementary Table 3). The instruments used are shown in Supplementary Tables 16–41. In our main analysis, inverse-variance-weighted (IVW) regression analysis, we found some strong evidence for a causal negative influence of DCC on MDD risk and a causal positive influence of DCC on LCU, the IVW regression odds ratio being 0.90 (95% CI: 0.85–0.95, *P* = 9.06 × 10^−5^) and 1.27 (95% CI: 1.11–1.46, *P* = 7.63 × 10^−4^), respectively. In the exploration of the causal effect of neuropsychiatric traits on DCC, we observed suggestive associations of higher genetically predicted AUDIT_T and AUDIT_C with DCC. For 1-SD increase in genetically predicted AUDIT_T and AUDIT_C, the OR was 1.83 (95% CI: 1.39–2.40, *P* = 1.67 × 10^−05^) and 1.80 (95% CI: 1.29–2.51, *P* = 5.09 × 10^−04^), respectively. Meanwhile, our results suggested that genetically predicted insomnia and MDD were negatively associated with DCC (OR: 0.91, 95% CI: 0.86–0.95, *P* = 1.51 × 10^−04^for Insomnia; OR: 0.93, 95% CI: 0.89–0.99, *P* = 6.02 × 10^−04^ for MDD). Besides, we found that there was no significant evidence for a causal relationship between DCC and other neuropsychiatric traits (Table 3, Supplementary Table 3). No significant horizontal pleiotropy and heterogeneity were detected in our last sensitivity analysis (Table 3). In addition, plots of the leave-one-out analysis supported that there was no potentially influential SNP driving the causal link and our conclusion was of stability (Supplementary Figures 6–29).

**Table 3:** The bidirectional MR analysis of decaffeinated coffee consumption and neuropsychiatric traits.

**Exposure-outcome**	**No. of SNP**	**IVW**				**MR egger**			
		**β**	***P*-Value**	***Q*-Value**	***P*-Value**	***Q*-Value**	***P*-Value**	**Intercept**	***P*-Value**
DCC-AD	31	−0.015	0.320	35.275	0.233	35.251	0.196	0.000	0.888
DCC-ALS	31	0.140	0.233	36.680	0.187	35.789	0.180	0.011	0.403
DCC-ADHD	27	0.059	0.553	33.290	0.154	32.803	0.136	0.006	0.548
DCC-BIP	31	0.005	0.949	26.947	0.626	26.278	0.611	0.007	0.420
DCC-AN	24	−0.001	0.995	29.425	0.167	28.962	0.146	−0.007	0.559
DCC-AUDIT_T	27	0.027	0.003	35.391	0.103	33.778	0.113	−0.001	0.285
DCC-AUDIT_C	28	0.014	0.057	29.985	0.315	29.357	0.295	−0.001	0.462
DCC-AUDIT_P	26	0.018	0.034	37.630	0.050	37.621	0.038	0.000	0.940
DCC-Insomnia	25	−0.040	0.303	32.412	0.117	32.411	0.092	0.000	0.983
DCC-LCU*	28	0.241	7.63E-04	30.858	0.277	30.644	0.242	0.003	0.673
DCC-MDD*	27	−0.107	9.06E-05	21.474	0.717	21.175	0.683	−0.002	0.590
DCC-Neuroticism	23	−0.041	0.109	59.736	0.000	58.145	0.000	−0.002	0.457
DCC-SCZ	11	−0.006	0.952	4.504	0.922	4.310	0.890	0.006	0.670
AD-DCC	76	0.002	0.956	92.985	0.078	92.813	0.069	0.001	0.712
ALS-DCC	21	−0.005	0.769	27.183	0.130	27.063	0.103	0.002	0.774
ADHD-DCC	86	−0.012	0.267	100.358	0.122	100.294	0.108	0.001	0.817
BIP-DCC	138	0.008	0.324	149.295	0.223	149.119	0.209	−0.001	0.689
AN-DCC	77	−0.015	0.204	88.495	0.155	87.278	0.157	−0.003	0.310
AUDIT_T-DCC*	62	0.603	1.67E-05	65.759	0.316	64.366	0.326	0.004	0.259
AUDIT_C-DCC*	51	0.589	5.09E-04	55.809	0.266	51.828	0.364	0.006	0.058
AUDIT_P-DCC	47	0.818	0.003	105.863	0.000	105.733	0.000	−0.001	0.816
Insomnia-DCC*	109	−0.099	1.51E-04	130.942	0.066	130.848	0.058	−0.001	0.782
LCU-DCC	64	0.019	0.171	59.735	0.593	54.238	0.748	0.005	0.022
MDD-DCC*	212	−0.077	6.02E-04	272.908	0.003	272.601	0.002	−0.001	0.627
Neuroticism-DCC	294	−0.088	0.008	354.888	0.008	353.747	0.008	0.002	0.333
SCZ-DCC	323	0.015	0.050	393.112	0.004	387.319	0.007	0.004	0.029

*IVW, inverse-variance weighted; DCC, decaffeinated coffee consumption; AD, Alzheimer's disease; ALS, Amyotrophic lateral sclerosis; ADHD, Attention deficit and hyperactivity disorder; BIP, bipolar disorder; AN, Anorexia nervosa; MDD, major depression disorder; LCU, lifetime cannabis use; SCZ, Schizophrenia; AUDIT_T, Alcohol use disorder identification test total score; AUDIT_C, alcohol consumption component of alcohol use disorder identification test item 1–3 score; AUDIT_P, dependence and hazardous-use component of alcohol use disorder identification test item 4–10 score*.

*Significant causality after Bonferroni correction (P <0.05/26 traits = 0.0019).

### Single-Trait TWAS

To evaluate the genes whose expression is related to DCC or neuropsychiatric traits and the common genes shared between those single-trait TWAS significant genes for DCC and neuropsychiatric traits, we performed a TWAS analysis. In total, we identified 74 gene-tissue pairs significantly associated with DCC across 48 GTEx tissues, in addition to 1,950, 10,802, 983, 5,876, 1,542, 1,400, and 96 gene-tissue pairs with insomnia, neuroticism, LCU, MDD, AUDIT_T, AUDIT_C, and AUDIT_P, respectively (Supplementary Tables 8–15). A large of TWAS-significant associations were identified in the brain, nerve, esophagus, colon, thyroid, testis, and adipose tissue, which are part of the nervous system, digestive system, and exo-/endocrine system. TWAS suggested that neuropsychiatric traits and DCC were regulated by a complex biological network. Among these associations, two gene-tissue pairs (CYP21A2-Brain Cerebellum, ZSCAN9-Brain Cerebellum) were overlapped in TWAS for DCC and MDD.

## Discussion

In the present study, we found strong genetic correlations between DCC and seven neuropsychiatric traits (insomnia, MDD, neuroticism, LCU, AUDIT_T, AUDIT_C, AUDIT_P) and identified common variants underlying these associations. Functional enrichment analysis was performed to suggest functions of the mapped genes. In addition, our bidirectional MR analysis suggested that DCC had a significant causal effect on an increased risk of LCU and a protective effect on MDD risk. Higher AUDIT_T and AUDIT_C may increase habitual DCC, but insomnia and MDD may decrease habitual DCC. These results advance our understanding of the common genetic structure between DCC and neuropsychiatric traits and reveal the causality between these traits, which demonstrated the shared etiologies and mechanisms in co-occurring DCC and neuropsychiatric traits.

Our cross-trait meta-analysis could capture the significant signals from both DCC and neuropsychiatric traits associated with GWAS summary statistics with great power. We identified 31 shared loci between DCC and neuroticism, 27 shared loci between DCC and MDD, six shared loci between DCC and insomnia, five shared loci between DCC and LCU, two shared loci between DCC and AUDIT_P, 1 shared locus between DCC and AUDIT_T, and 1 shared locus between DCC and AUDIT_C. Among them, 32 loci were not genome-wide significant in single-trait GWAS of both DCC and neuropsychiatric traits. Our results highlighted the important overlapped gene SLC39A8 (index SNP: rs13107325) identified by the cross-trait meta-analysis of seven genetically correlated trait pairs. Furthermore, our colocation analysis also showed that this locus had a great probability of containing shared causal variants of DCC and LCU (89%), as well as DCC and AUDIT (99%). SLC39A8 encodes a member of the SLC39 family of metal-ion transporters, which is glycosylated and found in the plasma membrane and mitochondria and involved in the cellular transport of zinc, the modulation of which could affect microglial inflammatory responses (50). Studies have reported that SLC39A8 was associated with caffeine consumption, alcohol consumption, and neuroticism (18, 51, 52). However, its contribution to LCU, insomnia, and MDD remains to be determined. The dysfunction of SLC39A8 had an impact on alterations in glutamate and immune function, for example, a reduction in GluN2A and GluA1/2/3 receptor surface expression, decreased BBB integrity, and increased IL-6/IL-1β protein expression (53), which may indicate that the occurrence of DCC and MDD, and LCU and insomnia is medicated by glutamate signaling and altered immune and inflammatory signals (54, 55).

Another overlapped gene TCF4, shared by three trait pairs (DCC/insomnia, DCC/neuroticism, and DCC/MDD), regulates the expression of cell adhesion molecules to control neuronal positioning during brain development (56). Polymorphisms in TCF4 were implicated in many psychiatric and neurological conditions (43). We also found several common variants (rs11214470 for LCU, rs867328 for neuroticism, rs4937872 for MDD) rarely reported in GWAS for DCC, LCU, neuroticism, and MDD. These variants were all mapped to NCAM1, encoding a cell adhesion protein. Secreted NCAM1 can bind to other extracellular molecules such as tenascin and the FGF receptor, all of which can form assemblies between different cells and guide cellular migration, neurogenesis, synaptic processes, and learning (46). So its worth further study to investigate how DCC and these three traits are similarly involved in synaptic plasticity, neurodevelopment, and neurogenesis. In addition, the strongest association in DCC/neuroticism meta-analysis was near to CELF4, expressed primarily in excitatory neurons. It regulates excitatory neurotransmission and binds to at least 15%−20% of transcriptomes, prominently playing in regulating and shaping the activity of neural circuits (45). Furthermore, our results found several genes or gene set significantly associated with DCC and MDD, including ZNFs (ZKSCAN4), BTNs (BTN1A1, BTN2A2, BTN3), and OR2B6. A large group of human ZNFs that contain a KRAB- and SCAN-box like ZKSCZN4 are thought to bind to DNA *via* their zinc finger domain, followed by transcriptional repression *via* the KRAB-box (48). BTNs are regulators of immune responses. Some of them such as BTN1A1 and BTN2A2 inhibited the proliferation of CD4^+^ and CD8^+^ T-cells and reduce the expression of IL-2 and INF-γ (57). BTN3A inhibited apoptosis for the increased survival of monocytes and dendritic cells, enhancing the synthesis of IL-1, IL-8, and IL-12. This is consistent with evidence showing the link between MDD and elevated proinflammatory cytokines (55, 58), as well as the association between coffee consumption and anti-inflammatory effects (23). Taken together, our results support that the genetic basis of DCC and neuropsychiatric traits are likely attributed to potential biological mechanisms of neurodevelopment, neural circuits, and immune response.

Our functional enrichment analysis provided several enriched tissues and biological pathways of the shared gene between DCC and neuropsychiatric traits involved. Tissue enrichment analysis showed that shared genes were enriched in nervous tissues (cerebellar cortex), exo-/endocrine tissues (thyroid gland), or hemic and immune tissues (Bone marrow, lymph node, tonsil), and lung. These findings provide clues for further study to investigate the potential common genetics in these enriched tissues. Reactome enrichment of HATs acetylate histones, HDACs deacetylate histones, HDMs demethylate histones, DNA methylation, and developmental biology together suggested the effects of both genetic and epigenetics on DCC/MDD and DCC/neuroticism. A study has revealed the shared pathway including DNA methylation between MDD and Insomnia (20). Inhibition of DNA methyltransferase 3a by polyphenol from coffee was also confirmed (59). These results imply the genetic and epigenetic contribution to both DCC and psychiatric disorders. Our GO analysis also showed pathway enrichment in the T cell receptor signaling pathway and antigen receptor-mediated signaling pathway. This is consistent with the anti-inflammatory effects of coffee and elevated proinflammatory cytokines of MDD (55, 58). Our functional analysis provides a new direction in studying epigenetic, developmental biology, or immune-related pathways in DCC and MDD or neuroticism.

In single-trait TWAS, large significant associations were identified in the brain, nerve, esophagus, colon, thyroid, testis, and adipose tissue, which are part of the nervous system, digestive system, and exo-/endocrine System. Our results suggested that neuropsychiatric traits and DCC were regulated by a complex biological network. Among these associations, two gene-tissue pairs (CYP21A2-Brain Cerebellum, ZSCAN9-Brain Cerebellum) were overlapped in TWAS of DCC and MDD. This suggested that studies need to pay more attention to the roles of the cerebellum region in DCC and MDD.

From bidirectional MR analysis, our findings indicated a causal influence of liability to DCC on LCU and low risk of MDD. The effects of coffee are commonly attributed to coffee's caffeine content. However, a study with 2,232 middle-aged men in Finland found no association between intake of caffeine and depression (60). A recent MR analysis also found little evidence to establish a causal relationship between caffeine assumption and cannabis use (61). Other compounds in coffee, such as chlorogenic acids and related compounds (quinines, caffeic acid), were found in amounts much more than that of caffeine and were thought to be responsible for antidepressant effects (62). These results provide novel insight into the role of compounds in decaffeinated coffee. On the other side, we found strong evidence for a causal relationship from high AUDIT_T and AUDIT_C to DCC but found no evidence for a causal relationship between DCC and AUDIT_P scores, confirming the different genetics between AUDIT subsets (63) and providing information for further study. Our results also suggested that insomnia and MDD had a causal negative influence on DCC. These may be explained by that the caffeine in caffeinated coffee acts primarily as a central nervous system stimulant, improving psychomotor performance, increasing vigilance, and reducing fatigue (21), which is what insomnia or MDD complainants look forward to. But it is worth drawing cautious conclusions on the influence of MDD on DCC because of the potential heterogeneity. Our MR findings provided evidence for relationships between DCC and neuropsychiatric traits, highlighting the important role of non-caffeine content in coffee. It also deserves further study to investigate whether and how caffeine affects or participates in the metabolism or mechanism of non-caffeine components in caffeinated coffee.

However, some limitations existed. First, lack of data on the amounts of compounds like chlorogenic acids present in the decaffeinated coffee regularly consumed by study participants. But qualitative research still can provide strong evidence to some extent. Second, the GWAS data from meta-analysis studies have great power but reduces the homogeneity. Fortunately, the method CPASSOC can deal with population structure and cryptic relatedness.

In conclusion, we found strong genetic correlations between DCC and neuropsychiatric disorders and identified shared common variants underlying these associations, suggesting a common genetic influence across these traits. Functional analysis showed epigenetic, developmental biology, or immune-related pathways in DCC and MDD or neuroticism, providing new directions in common biology process studies. MR analysis found strong evidence for a causal relationship between DCC and six neuropsychiatric traits (insomnia, MDD, LCU, AUDIT_T, AUDIT_C, AUDIT_P). These findings advance our understanding of the shared genetic mechanisms and causality underlying their associations, assisting with making recommendations for clinical works or health education.

## Data Availability Statement

The original contributions presented in the study are included in the article/Supplementary Material, further inquiries can be directed to the corresponding authors.

## Author Contributions

BY performed the statistical analysis and wrote the manuscript. All authors designed the study, helped interpret the data, reviewed and edited the final paper, and approved the submission.

## Funding

The study was supported by grants from the Peking University Start-up Grant (BMU2018YJ002), the High-performance Computing Platform of Peking University. The funding organization had no role in the preparation of the manuscript.

## Conflict of Interest

The authors declare that the research was conducted in the absence of any commercial or financial relationships that could be construed as a potential conflict of interest.

## Publisher's Note

All claims expressed in this article are solely those of the authors and do not necessarily represent those of their affiliated organizations, or those of the publisher, the editors and the reviewers. Any product that may be evaluated in this article, or claim that may be made by its manufacturer, is not guaranteed or endorsed by the publisher.
