# Supplementary material for: Shared Genetics and Causality Between Decaffeinated Coffee Consumption and Neuropsychiatric Diseases: A Large-Scale Genome-Wide Cross-Trait Analysis and Mendelian Randomization Analysis
Source: Front Psychiatry. 2022 Jul 11;13:910432. doi: 10.3389/fpsyt.2022.910432 (PMC9309364; doi:10.3389/fpsyt.2022.910432)
Supplement: Supplementary file 2 [file Data_Sheet_2.docx]

Supplementary Material

# Supplementary Figures and Tables

## Supplementary Figures

**
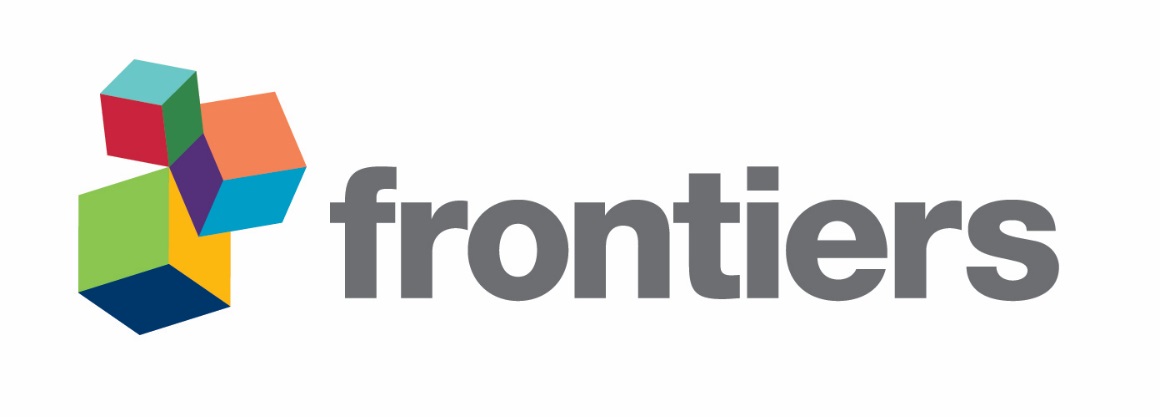
**

**Supplementary Figure 1.** Tissue enrichment analysis result of shared genes between decaffeinated coffee consumption and neuroticism


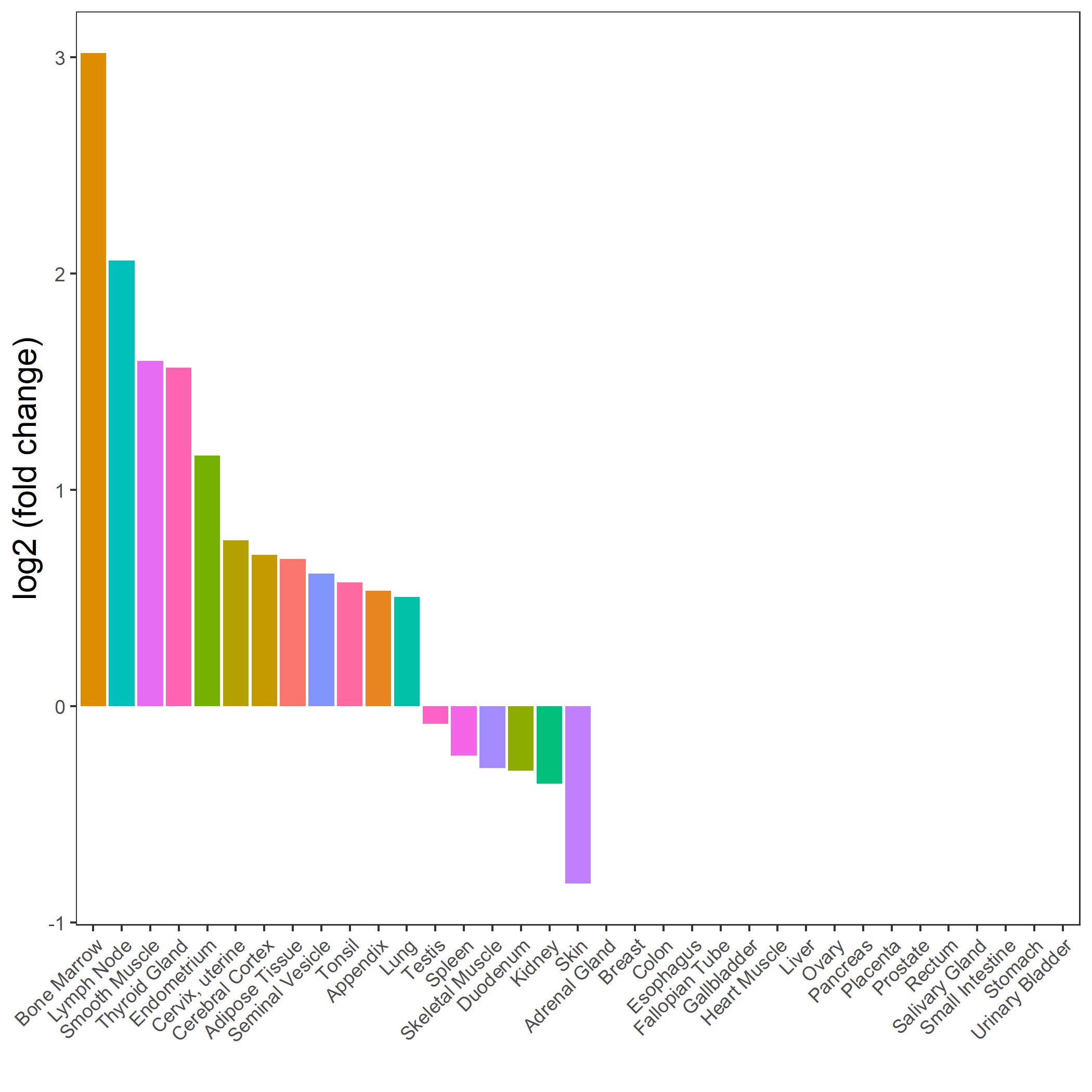


**Supplementary Figure 2.** Tissue enrichment analysis result of shared genes between decaffeinated coffee consumption and MDD


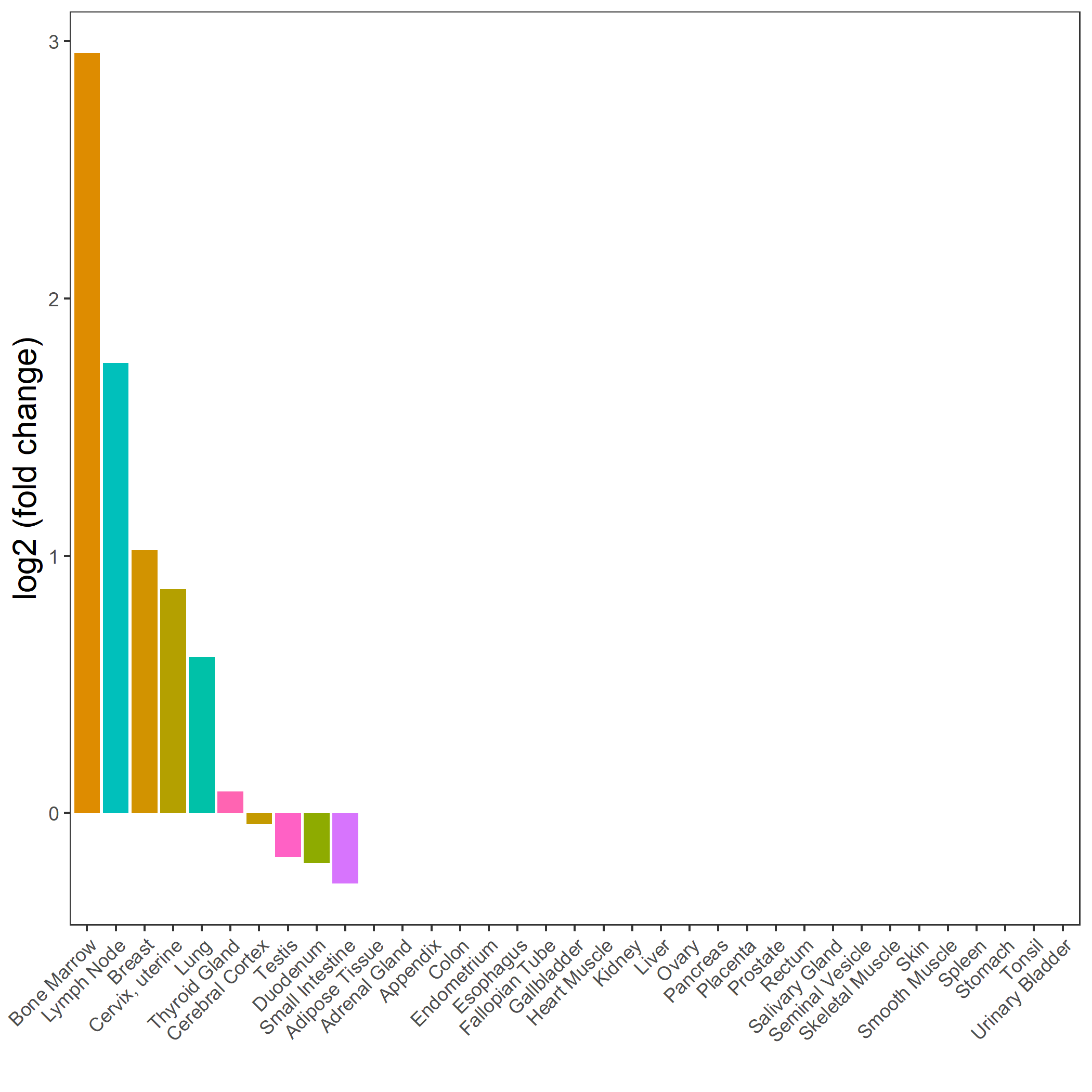


**Supplementary Figure 3.** Tissue enrichment analysis result of shared genes between decaffeinated coffee consumption and LCU
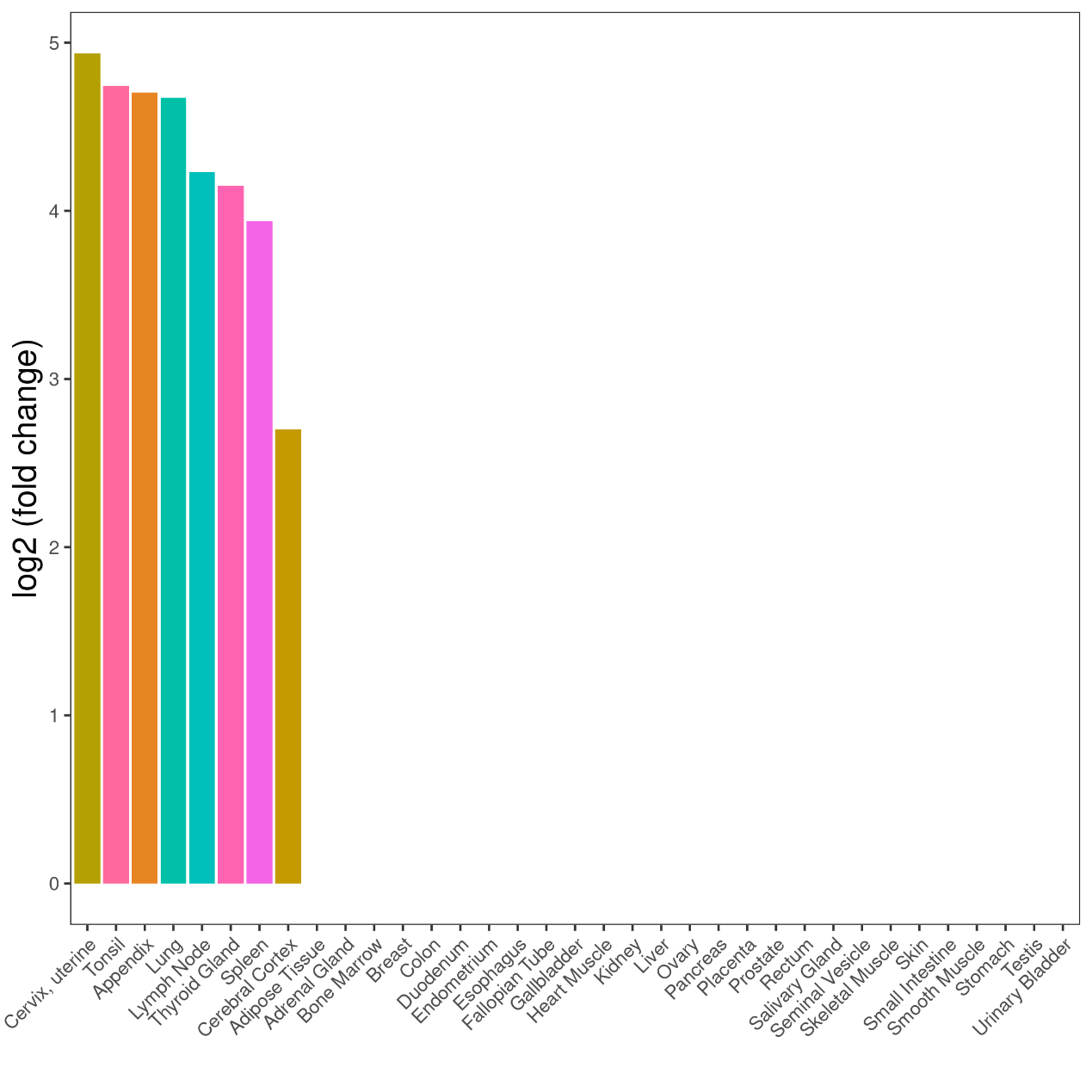


**Supplementary Figure 4.** Tissue enrichment analysis result of shared genes between decaffeinated coffee consumption and insomnia


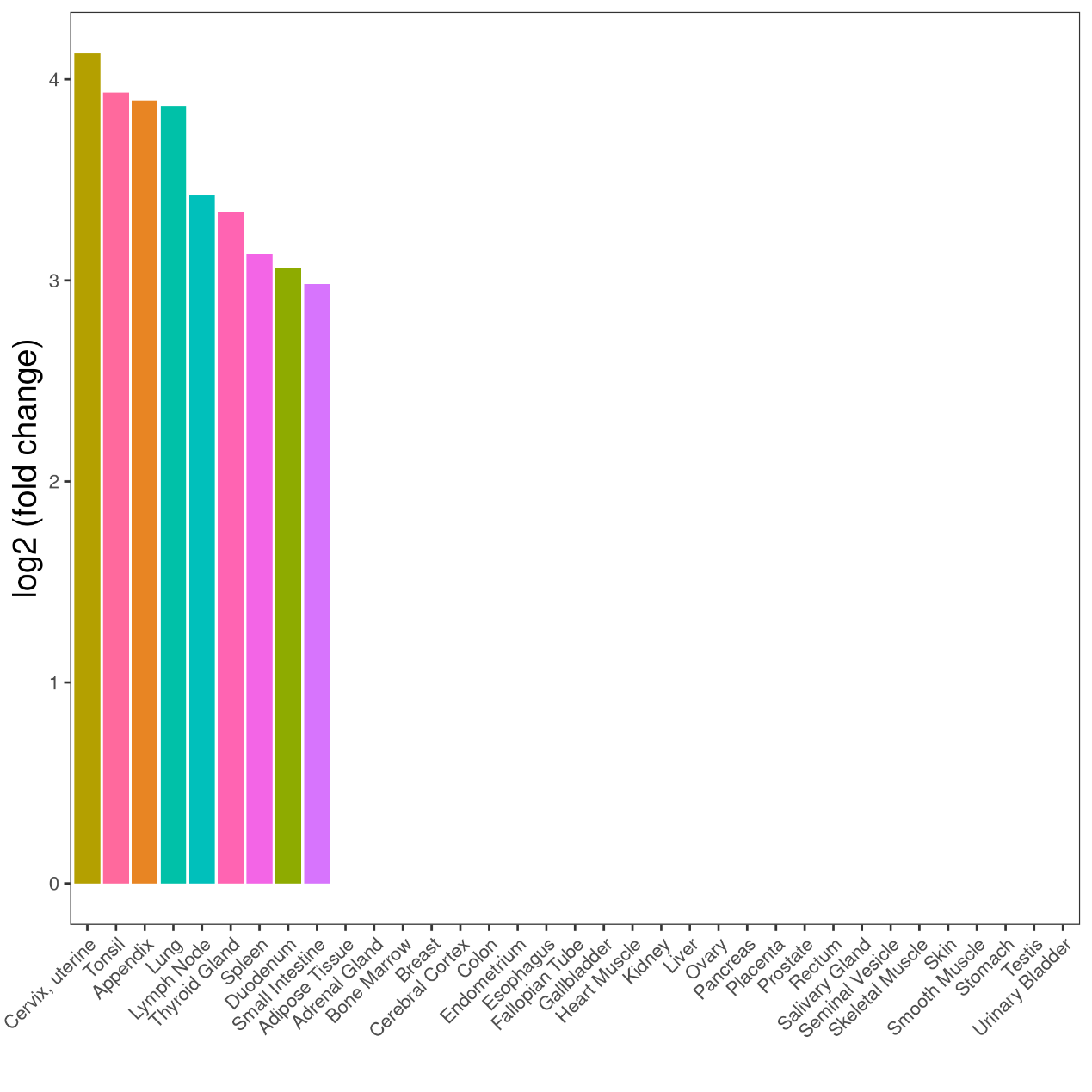


**Supplementary Figure 5.** Tissue enrichment analysis result of shared genes between decaffeinated coffee consumption and AUDIT_T/AUDIT_C/AUDIT_P


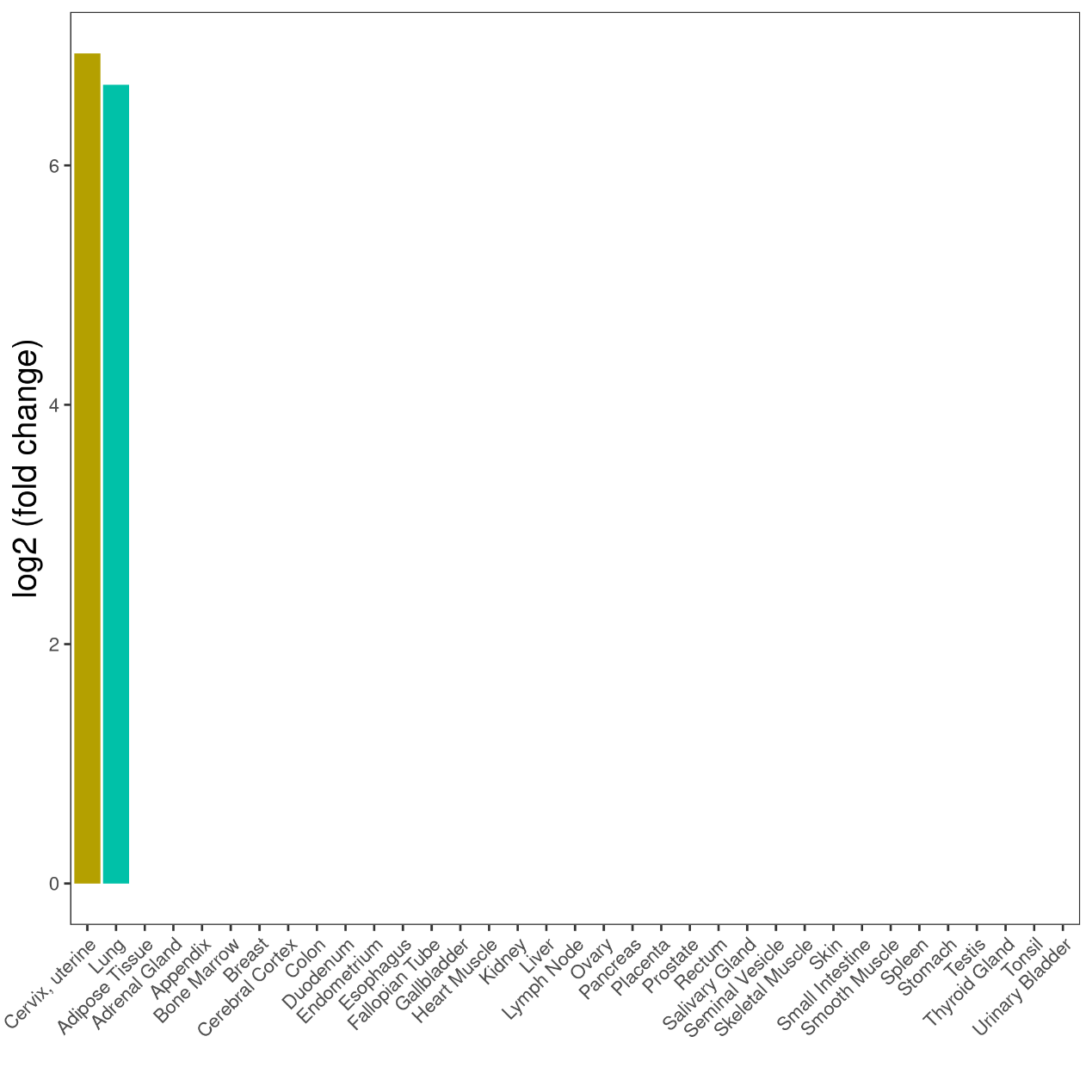


**Supplementary Figure 6.** Scatter plot of MR analysis of decaffeinated coffee consumption upon LCU


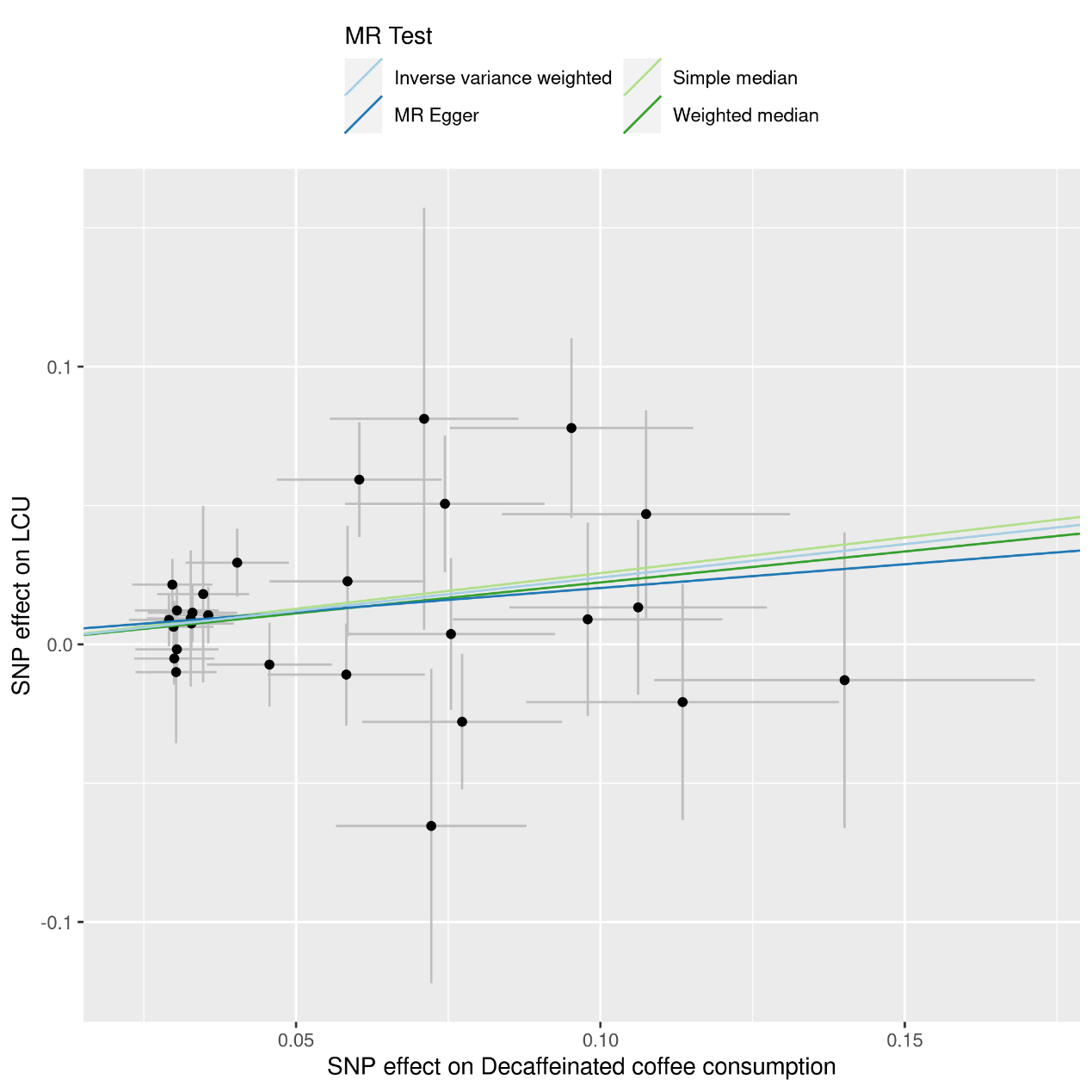


**Supplementary Figure 7.** Scatter plot of MR analysis of decaffeinated coffee consumption upon MDD


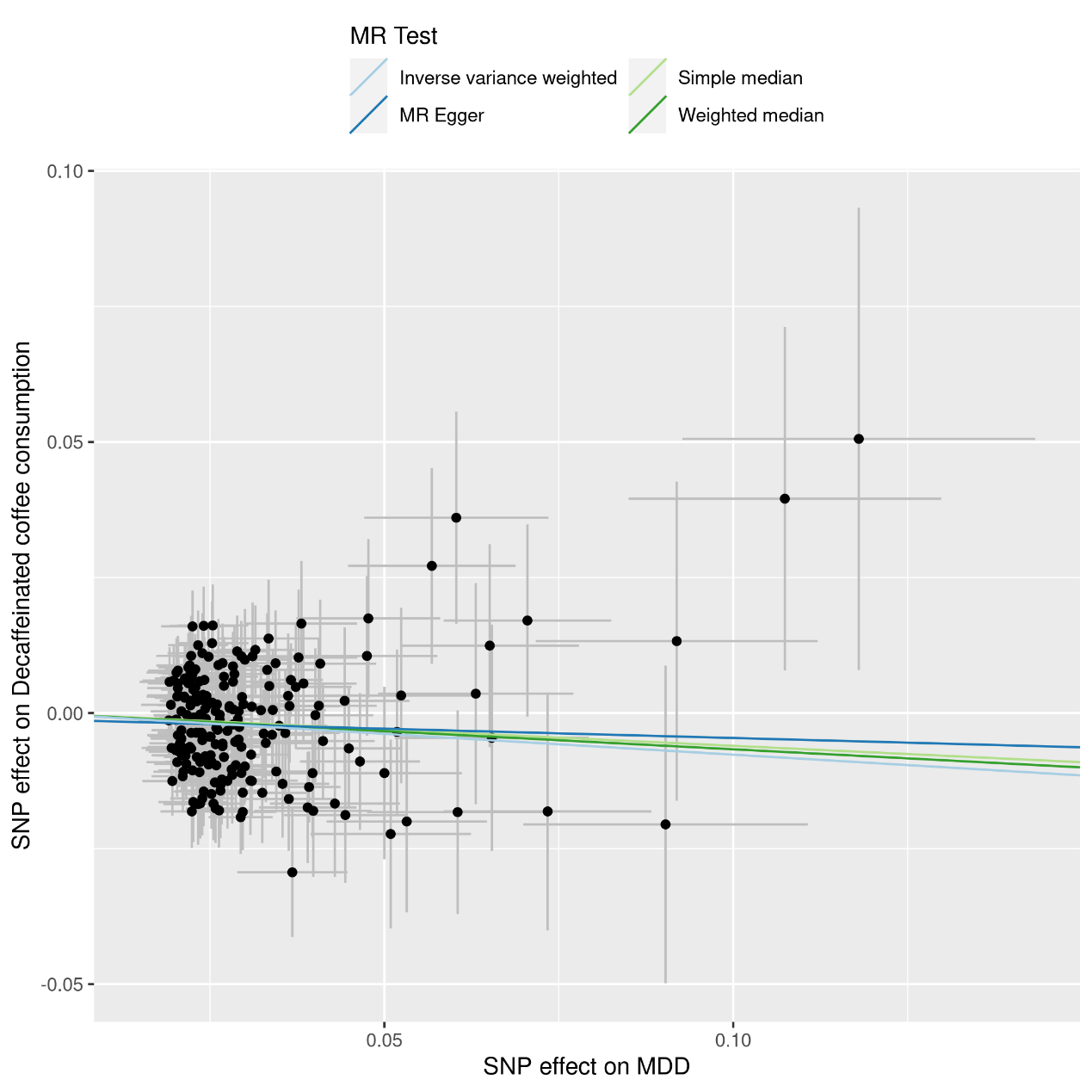


**Supplementary Figure 8.** Scatter plot of MR analysis of AUDIT_T upon decaffeinated coffee consumption


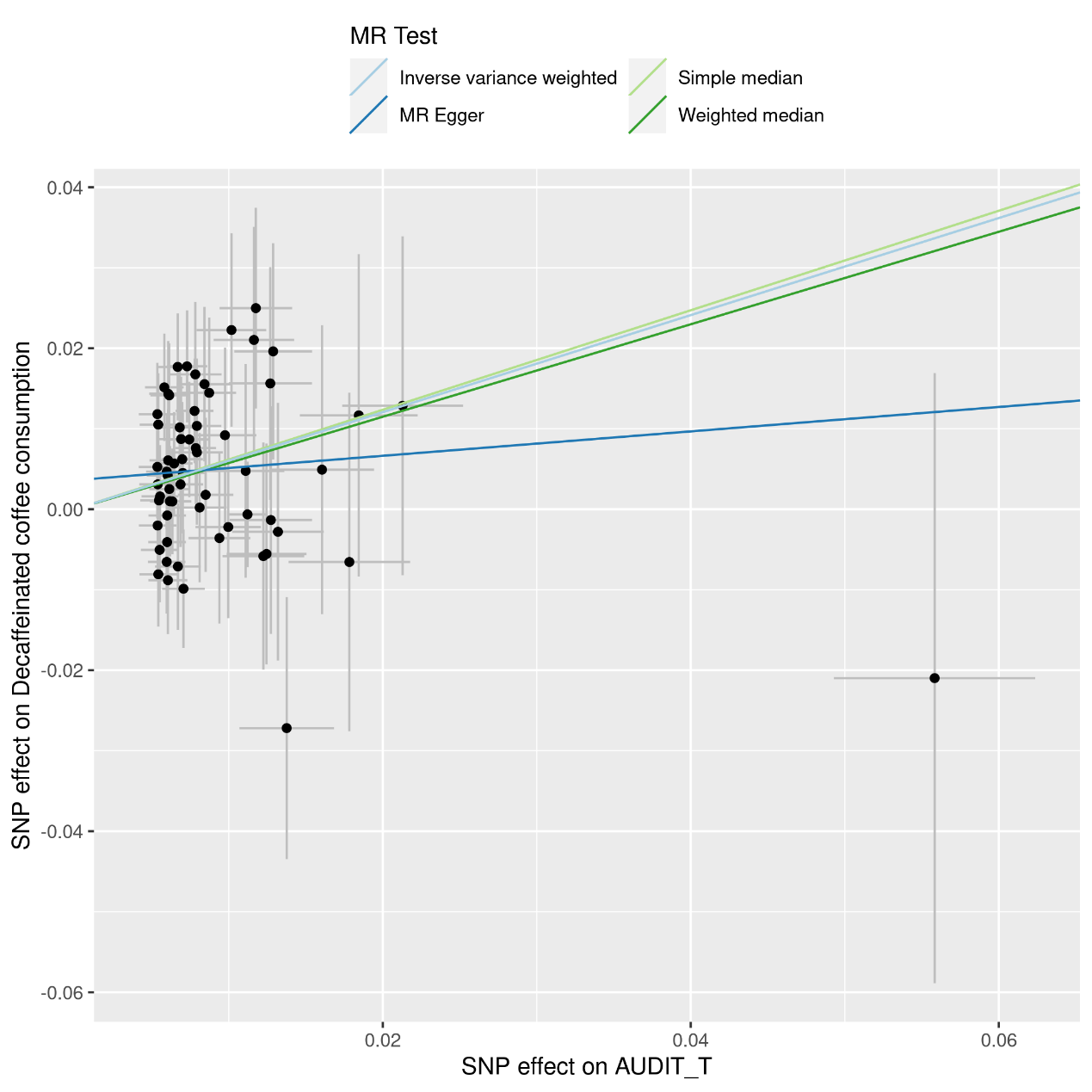


**Supplementary Figure 9.** Scatter plot of MR analysis of AUDIT_C upon decaffeinated coffee consumption


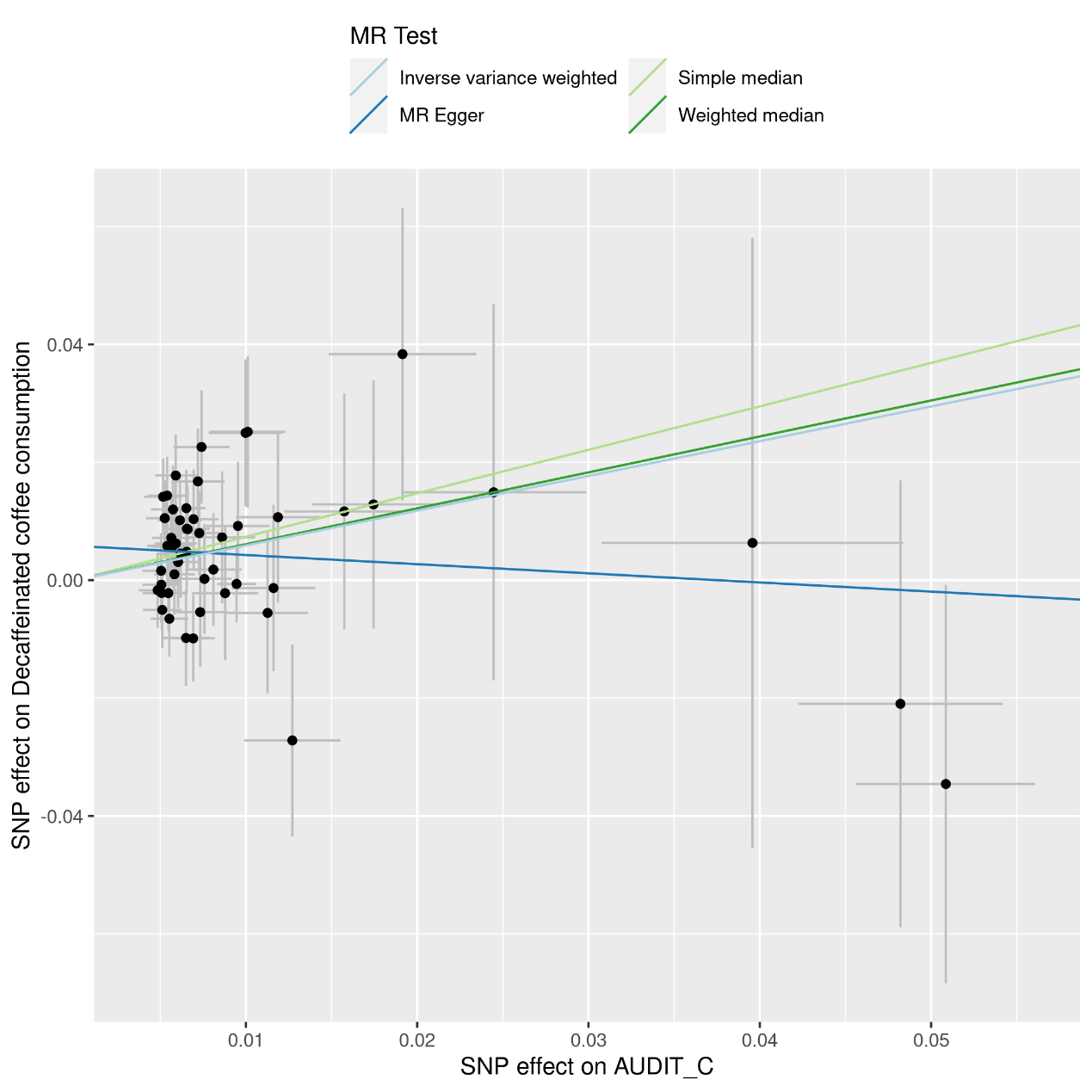


**Supplementary Figure 10.** Scatter plot of MR analysis of Insomnia upon decaffeinated coffee consumption


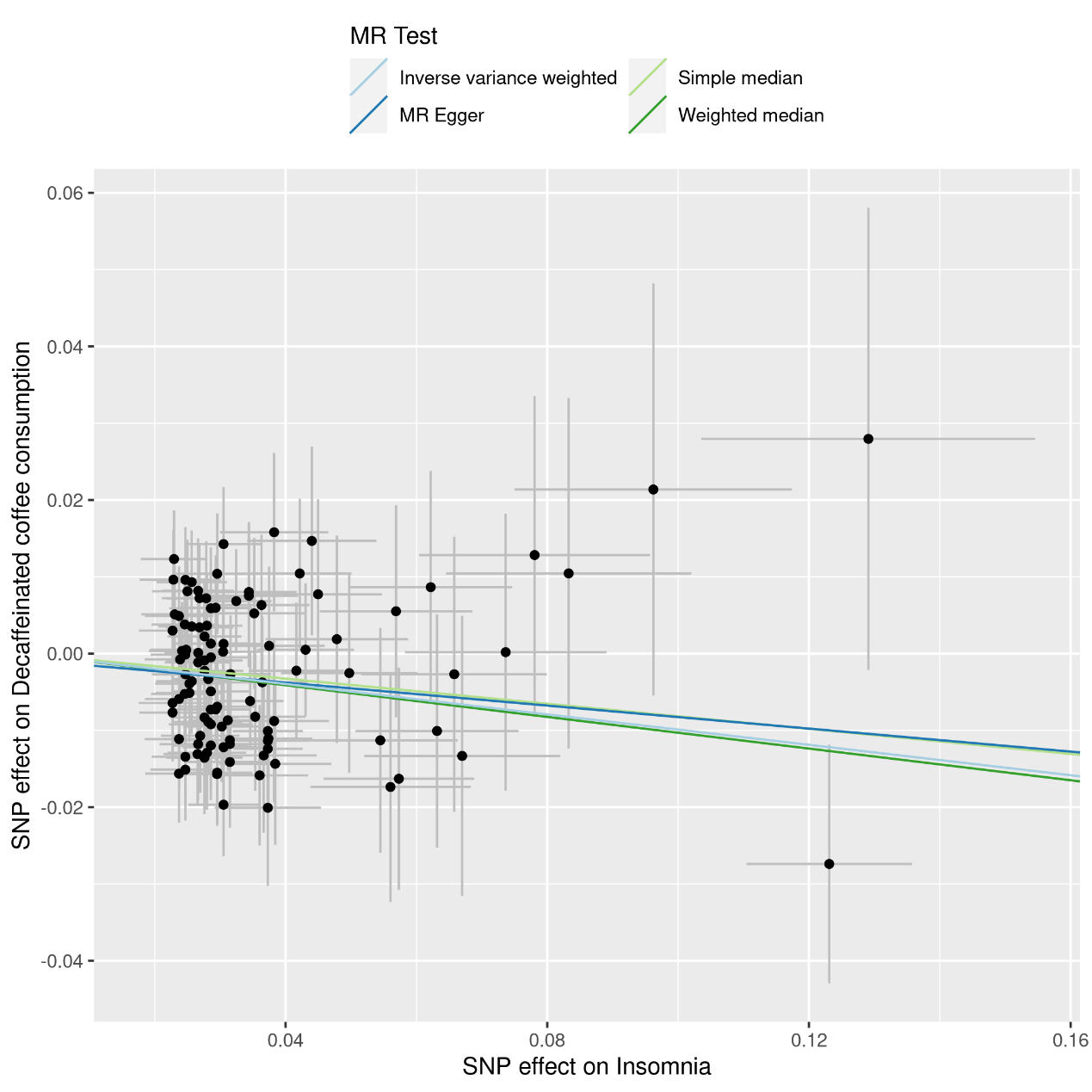


**Supplementary Figure 11.** Scatter plot of MR analysis of MDD upon decaffeinated coffee consumption


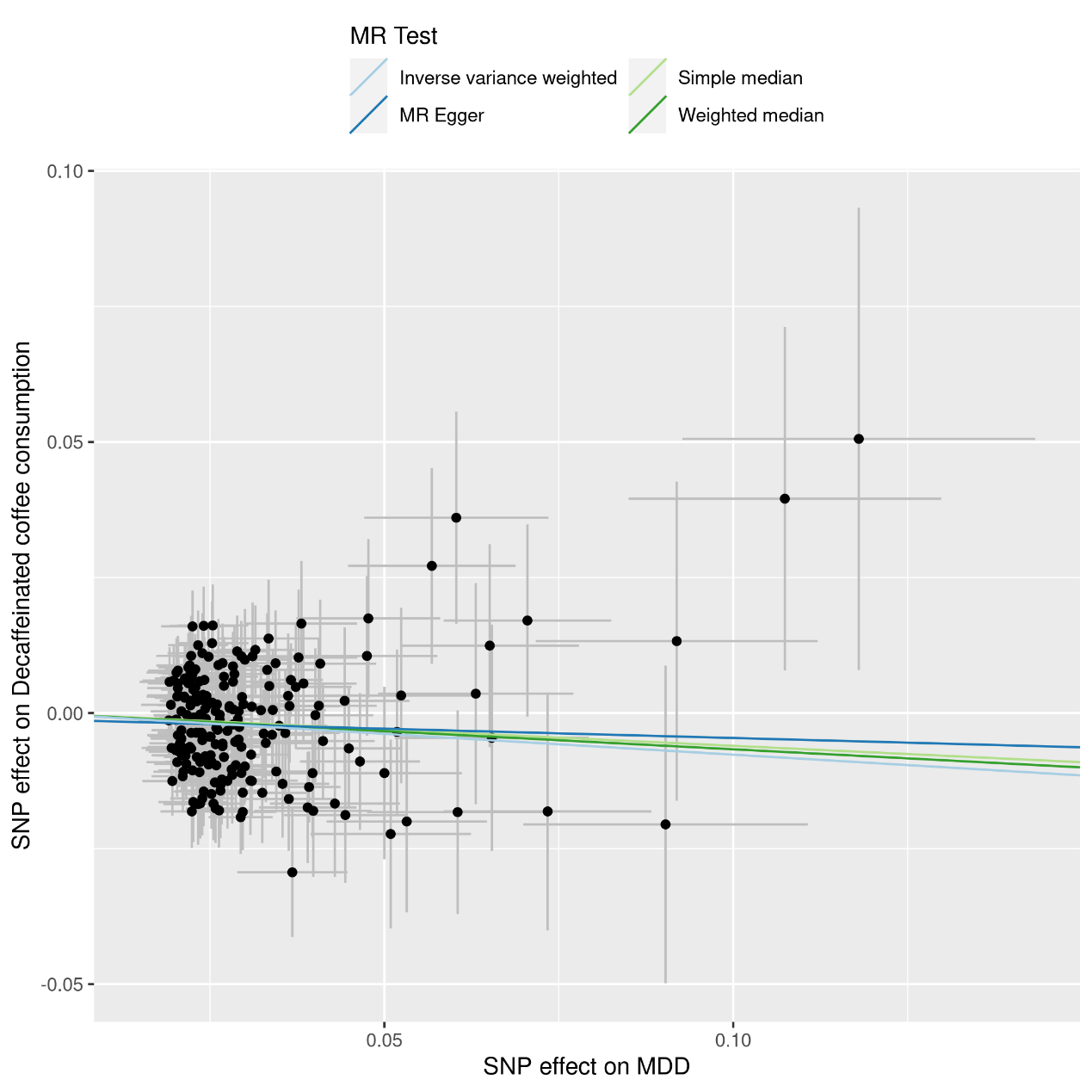


**Supplementary Figure 12.** Funnel plot of MR analysis of decaffeinated coffee consumption upon LCU


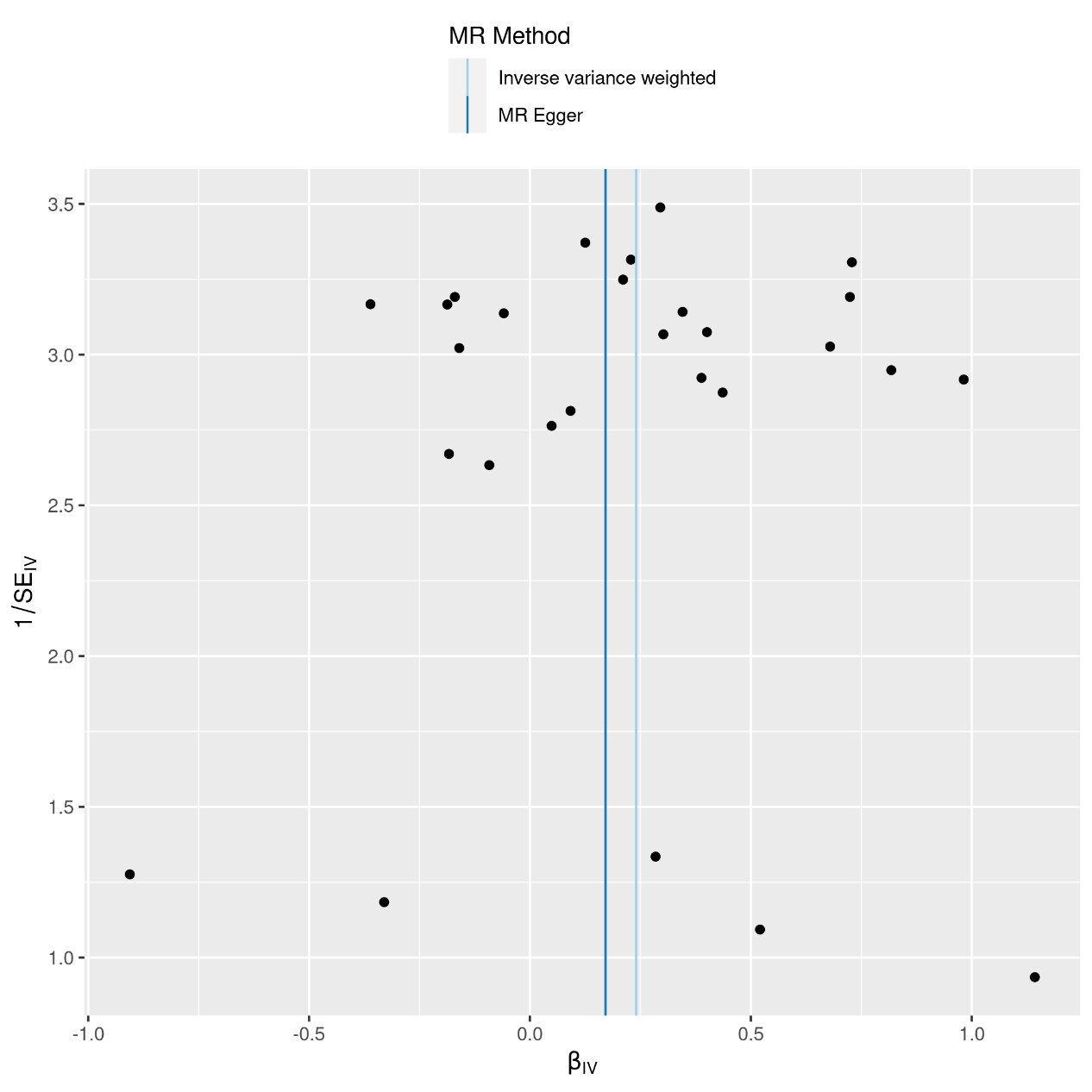


**Supplementary Figure 13.** Funnel plot of MR analysis of decaffeinated coffee consumption upon MDD


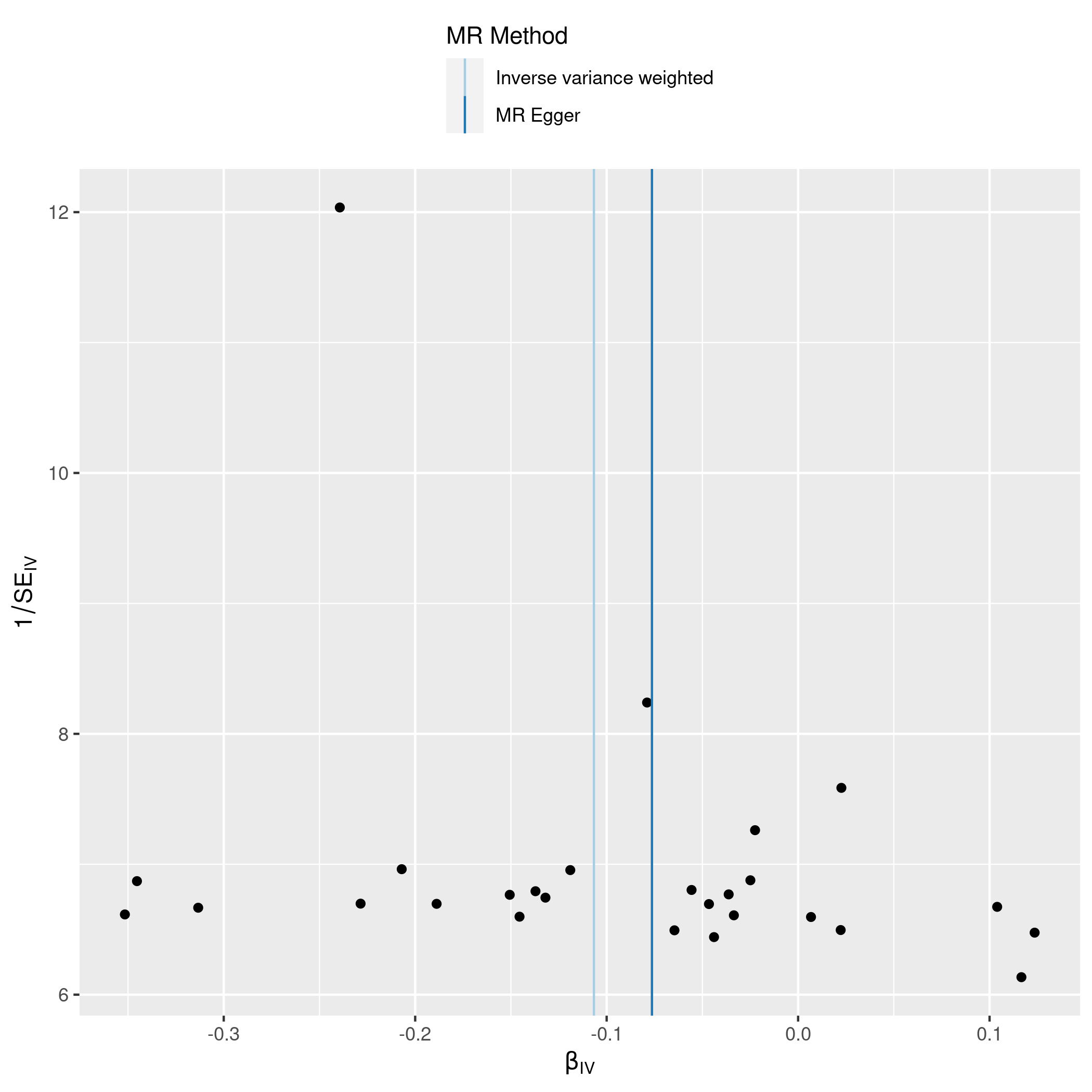


**Supplementary Figure 14.** Funnel plot of MR analysis of AUDIT_T upon decaffeinated coffee consumption


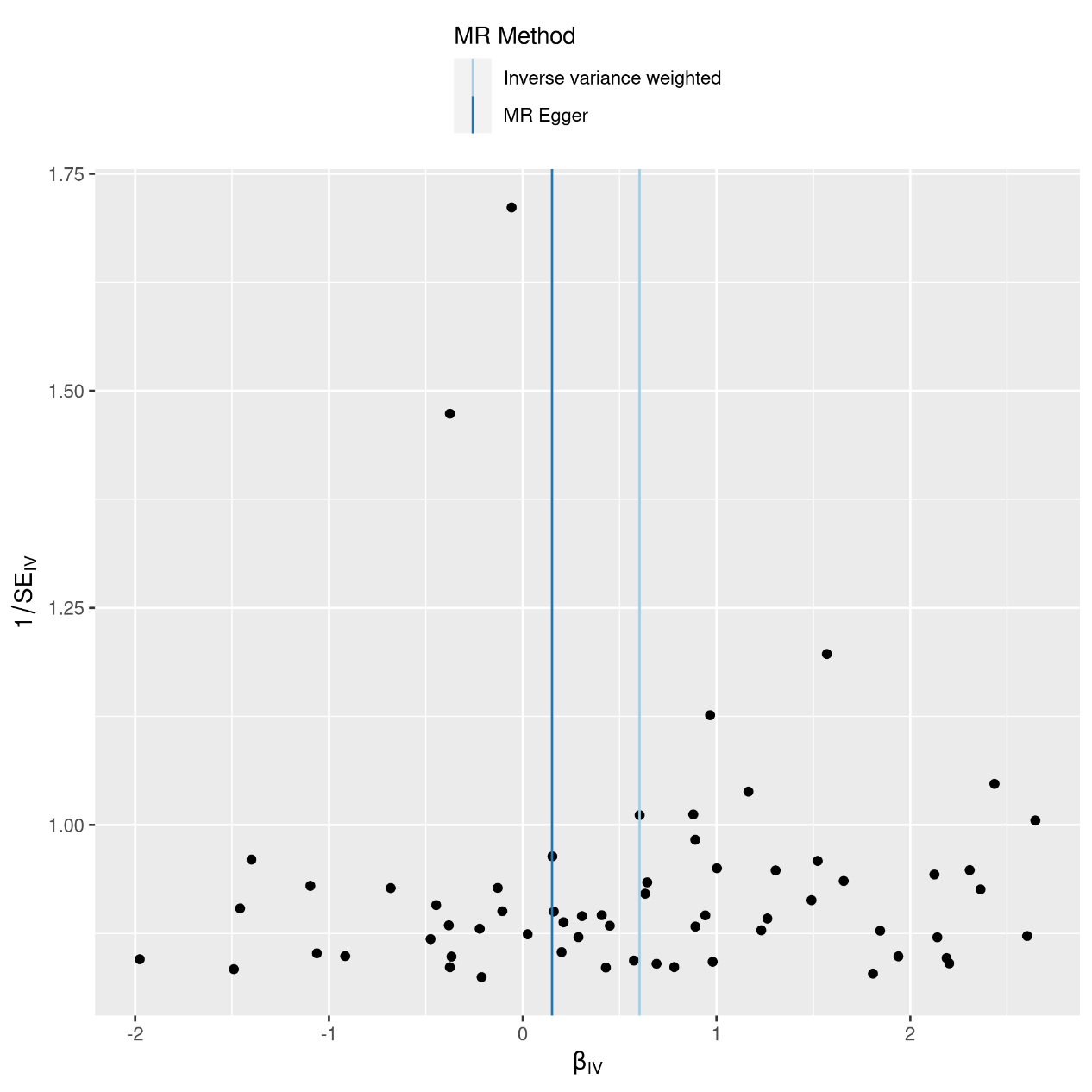


**Supplementary Figure 15.** Funnel plot of MR analysis of AUDIT_C upon decaffeinated coffee consumption


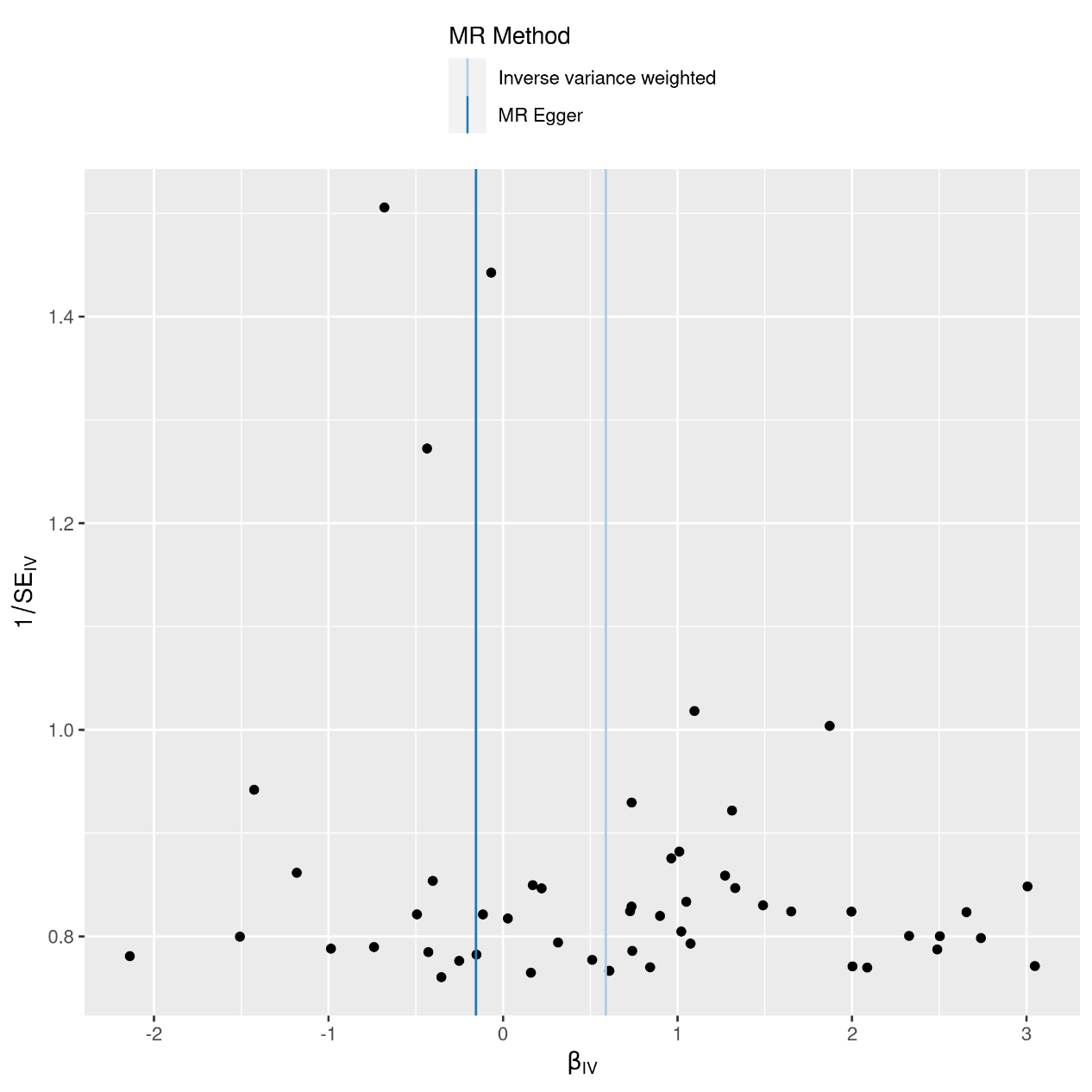


**Supplementary Figure 16.** Funnel plot of MR analysis of Insomnia upon decaffeinated coffee consumption


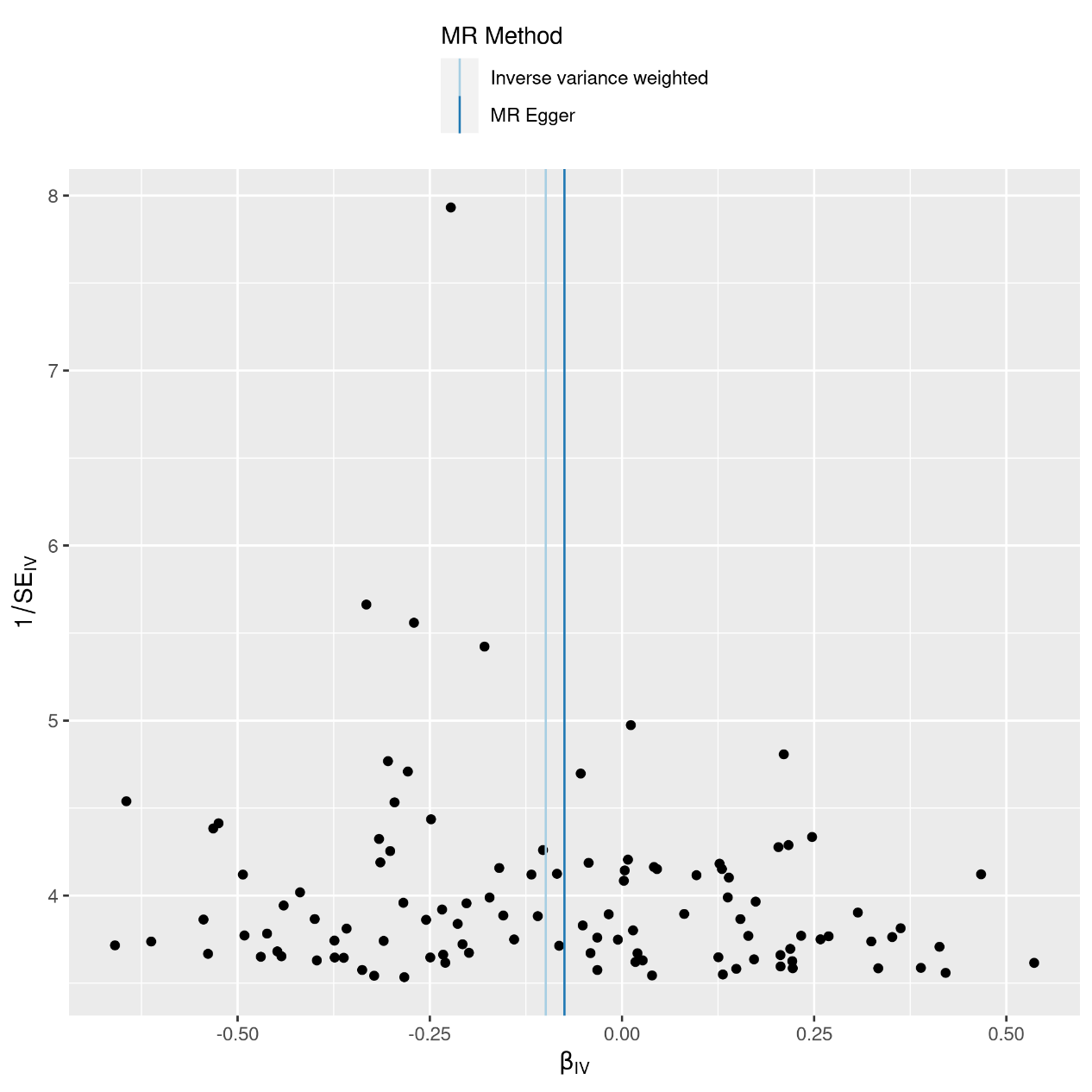


**Supplementary Figure 17.** Funnel plot of MR analysis of MDD upon decaffeinated coffee consumption


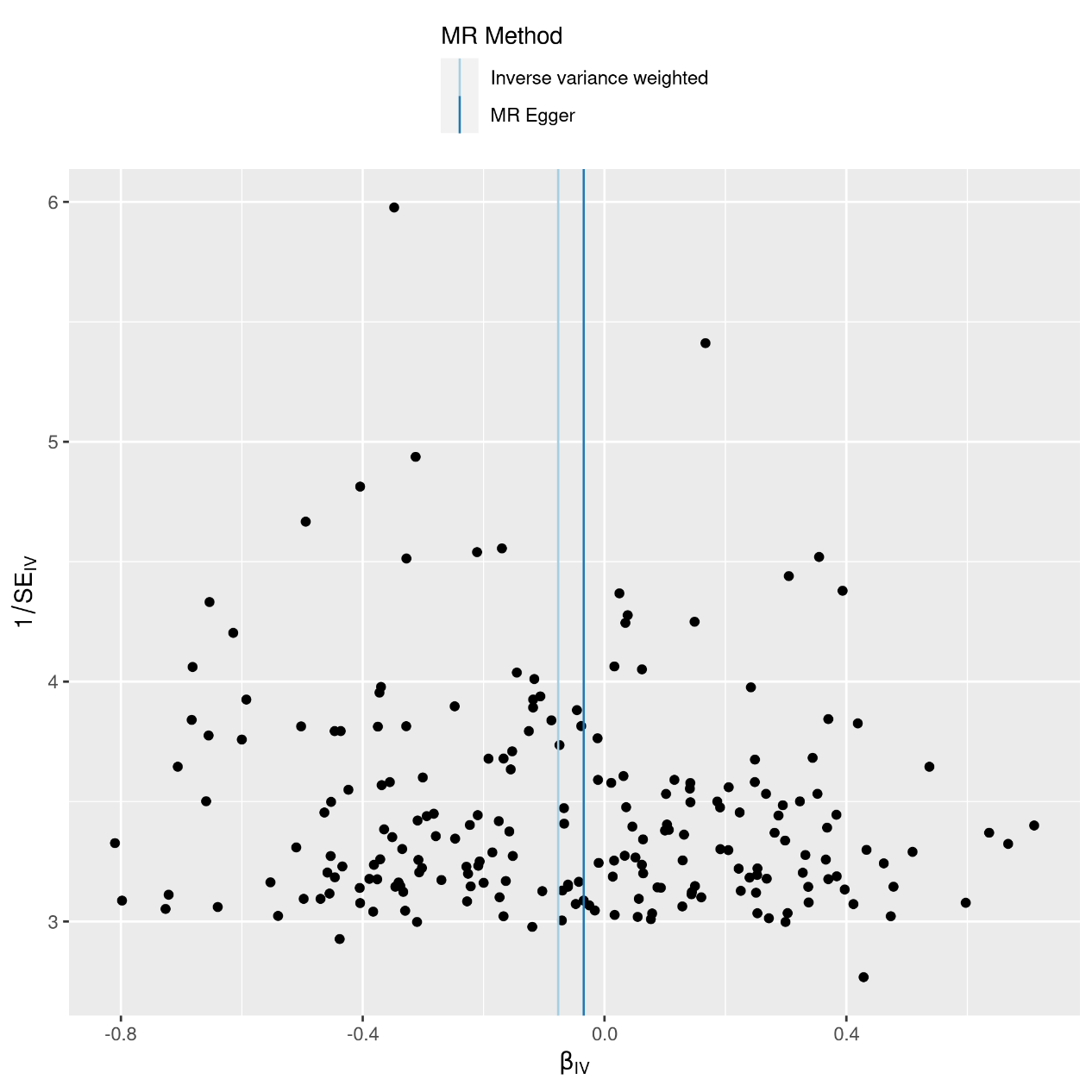


**Supplementary Figure 18.** Leave-one-out plot of MR analysis of decaffeinated coffee consumption upon LCU


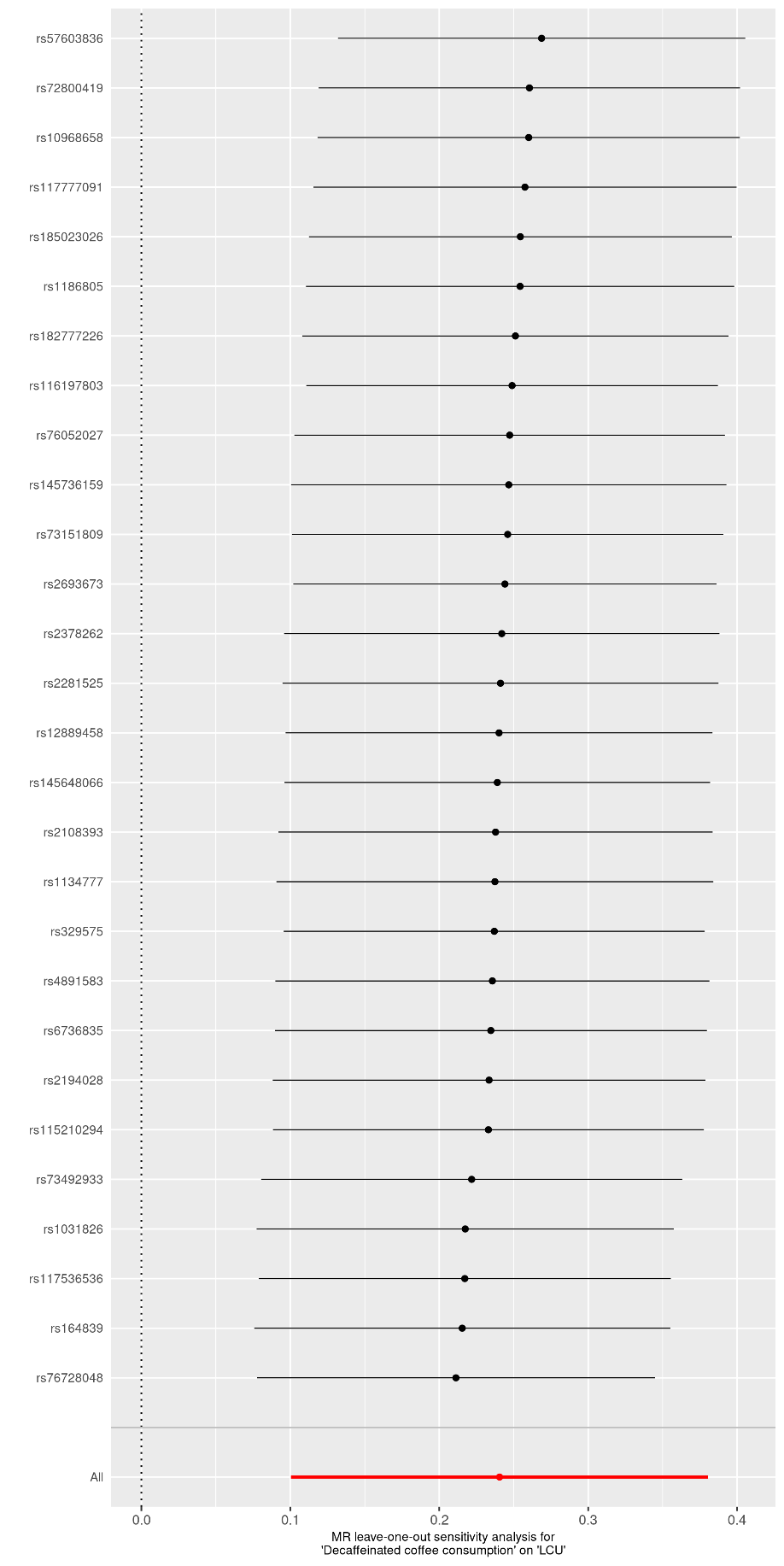


**Supplementary Figure 19.** Leave-one-out plot of MR analysis of decaffeinated coffee consumption upon MDD


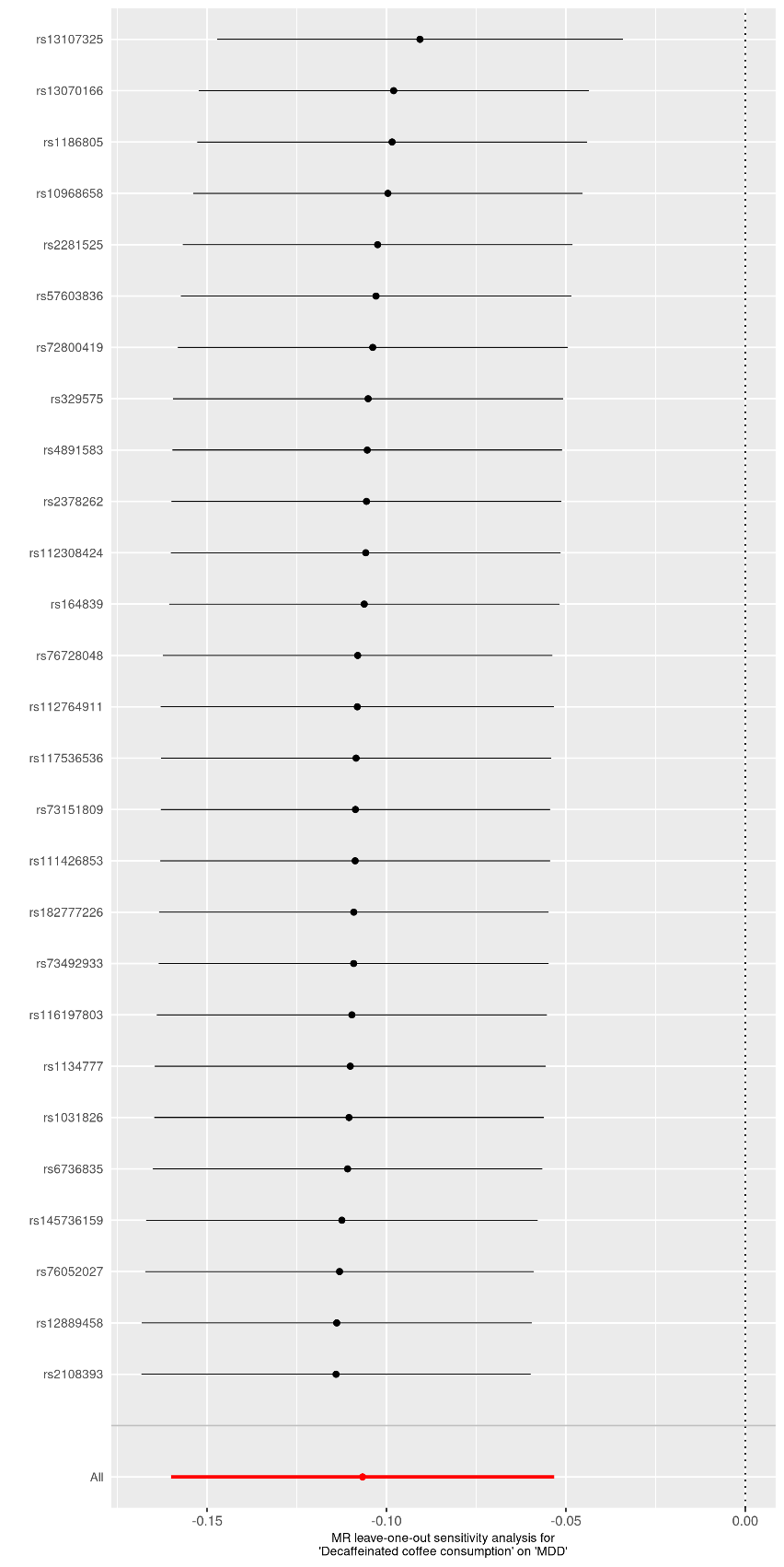


**Supplementary Figure 20.** Leave-one-out plot of MR analysis of AUDIT_T upon decaffeinated coffee consumption


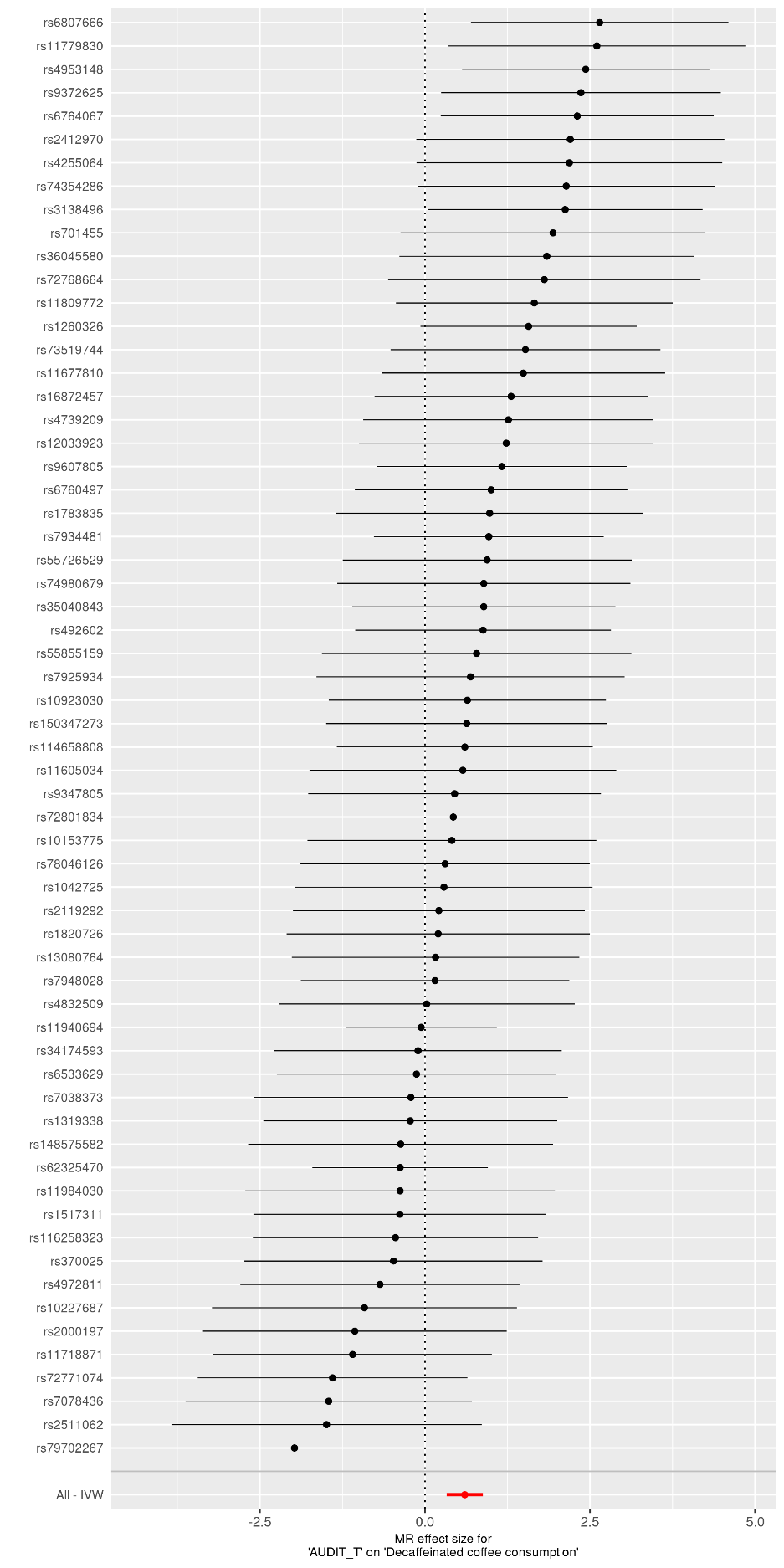


**Supplementary Figure 21.** Leave-one-out plot of MR analysis of AUDIT_C upon decaffeinated coffee consumption


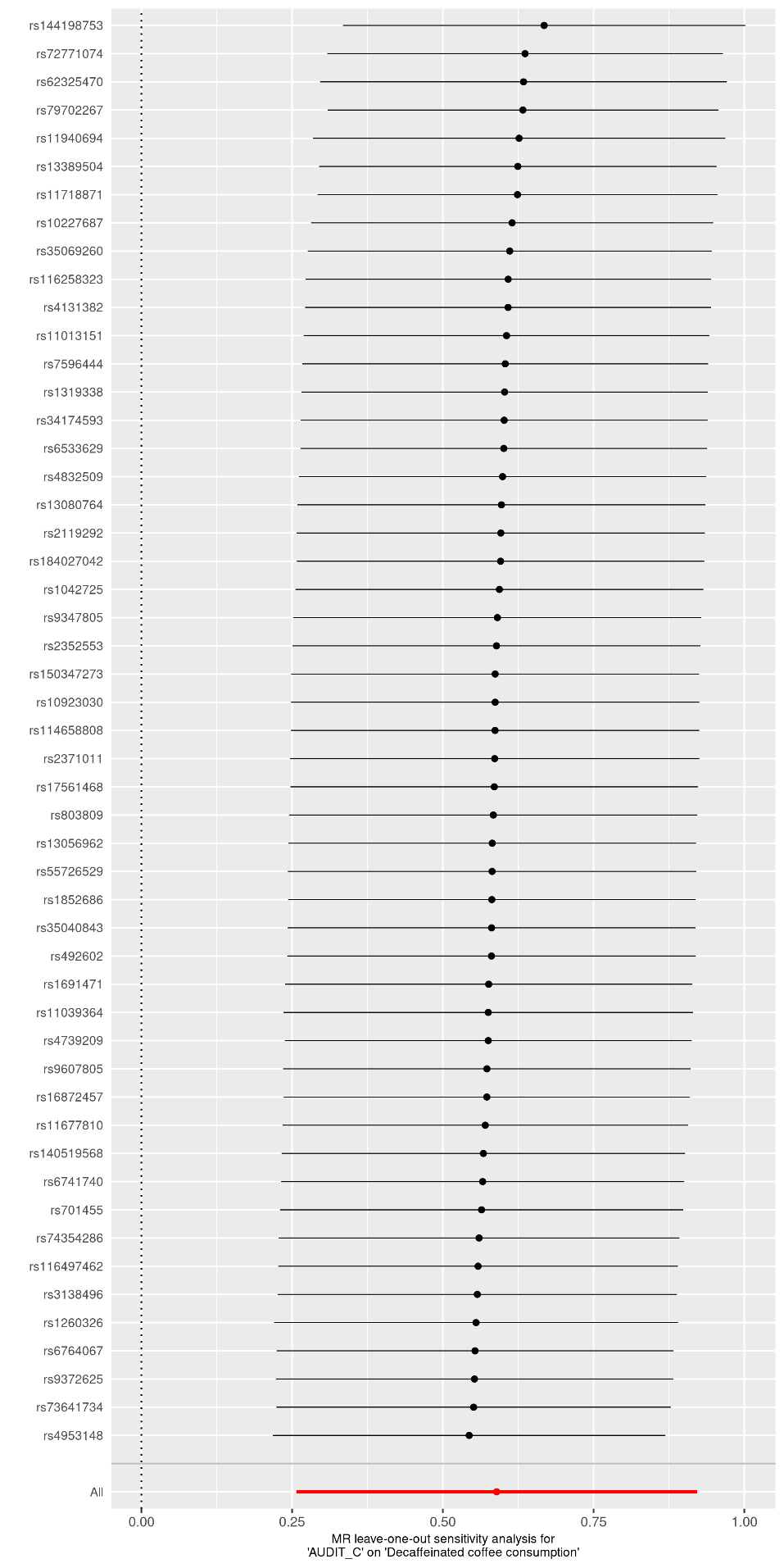


**Supplementary Figure 22.** Leave-one-out plot of MR analysis of Insomnia upon decaffeinated coffee consumption


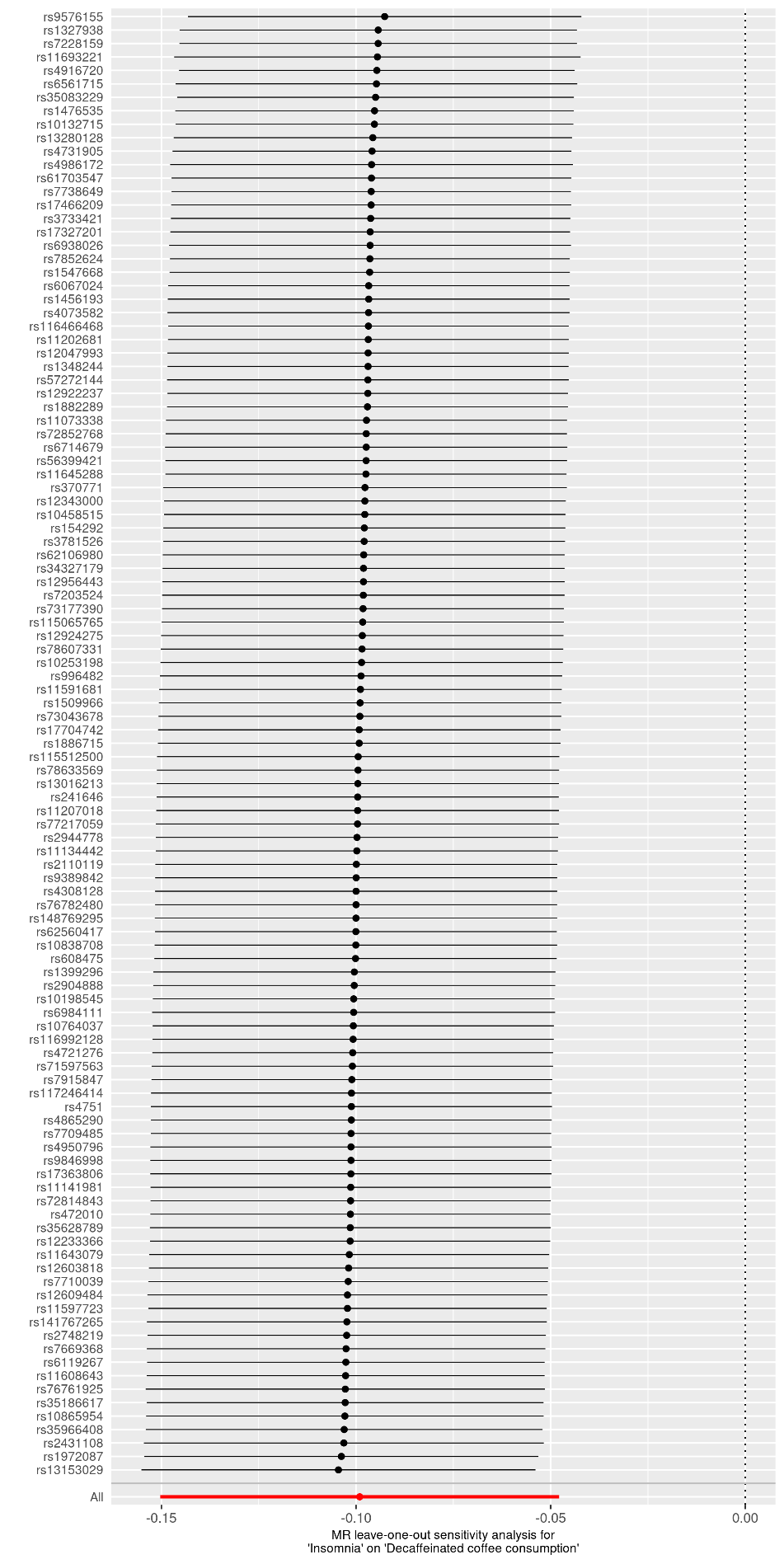


**Supplementary Figure 23.** Leave-one-out plot of MR analysis of MDD upon decaffeinated coffee consumption


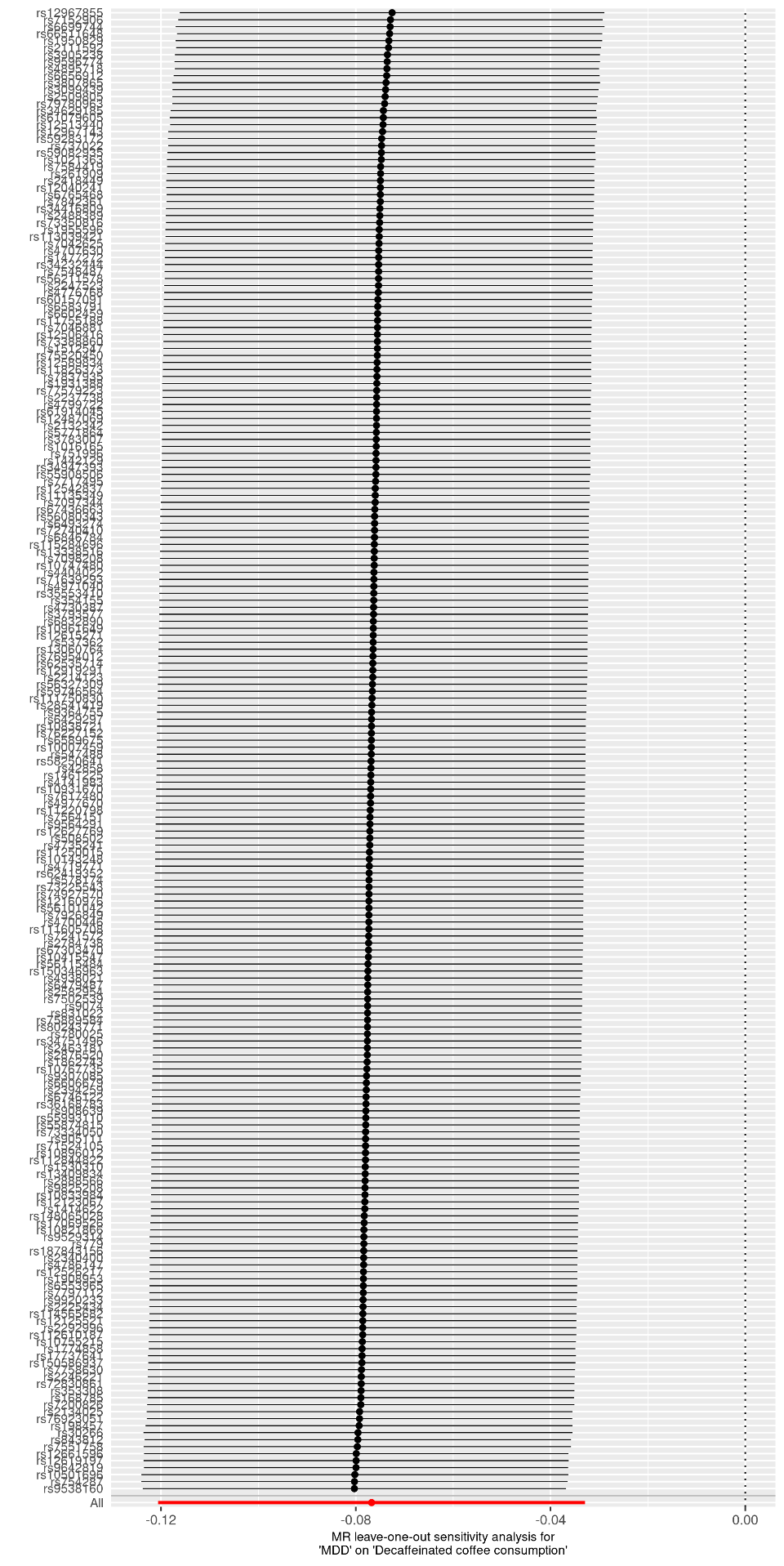


**Supplementary Figure 24.** Forest plot of MR analysis of decaffeinated coffee consumption upon LCU


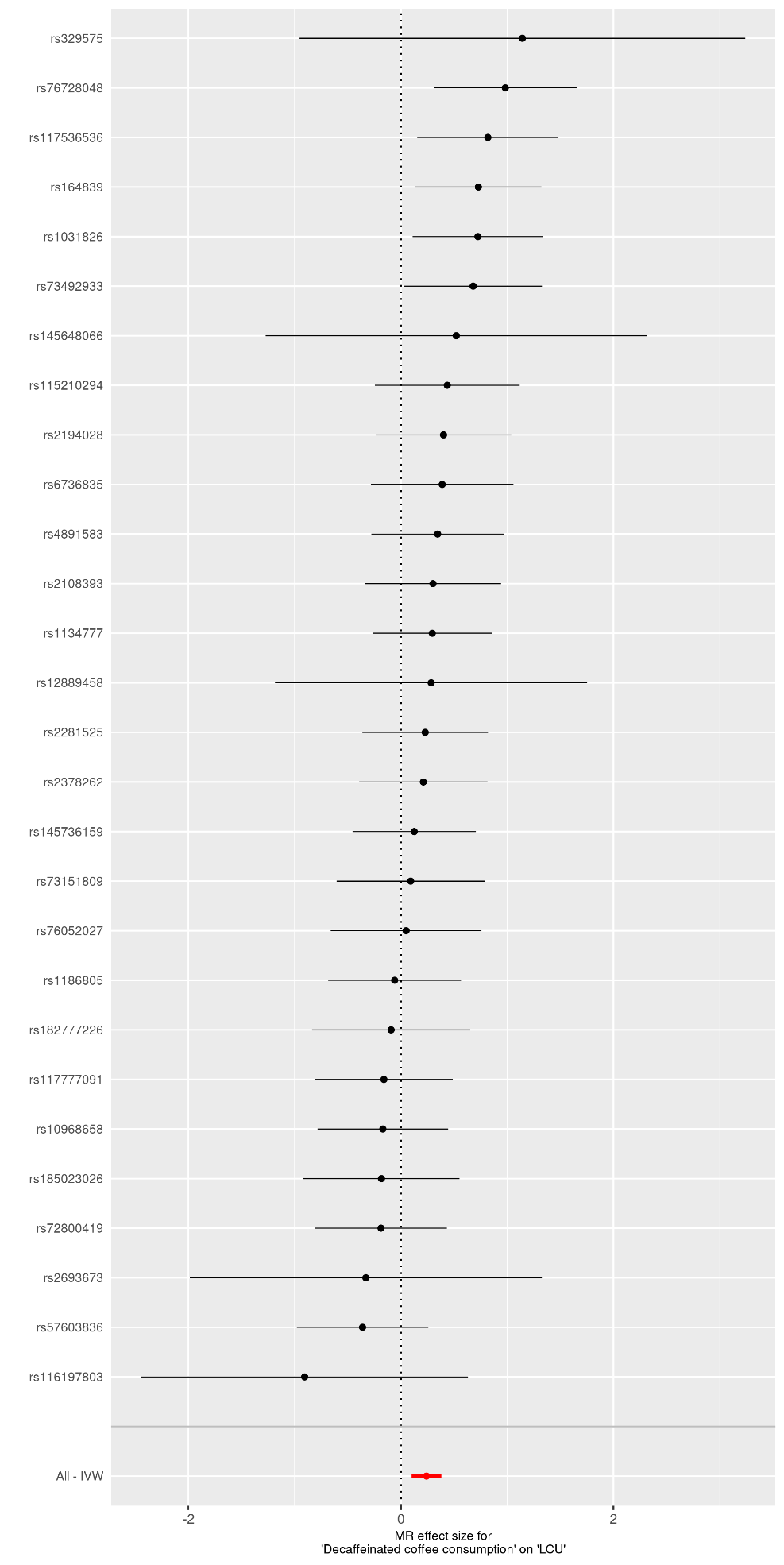


**Supplementary Figure 25.** Forest plot of MR analysis of decaffeinated coffee consumption upon MDD


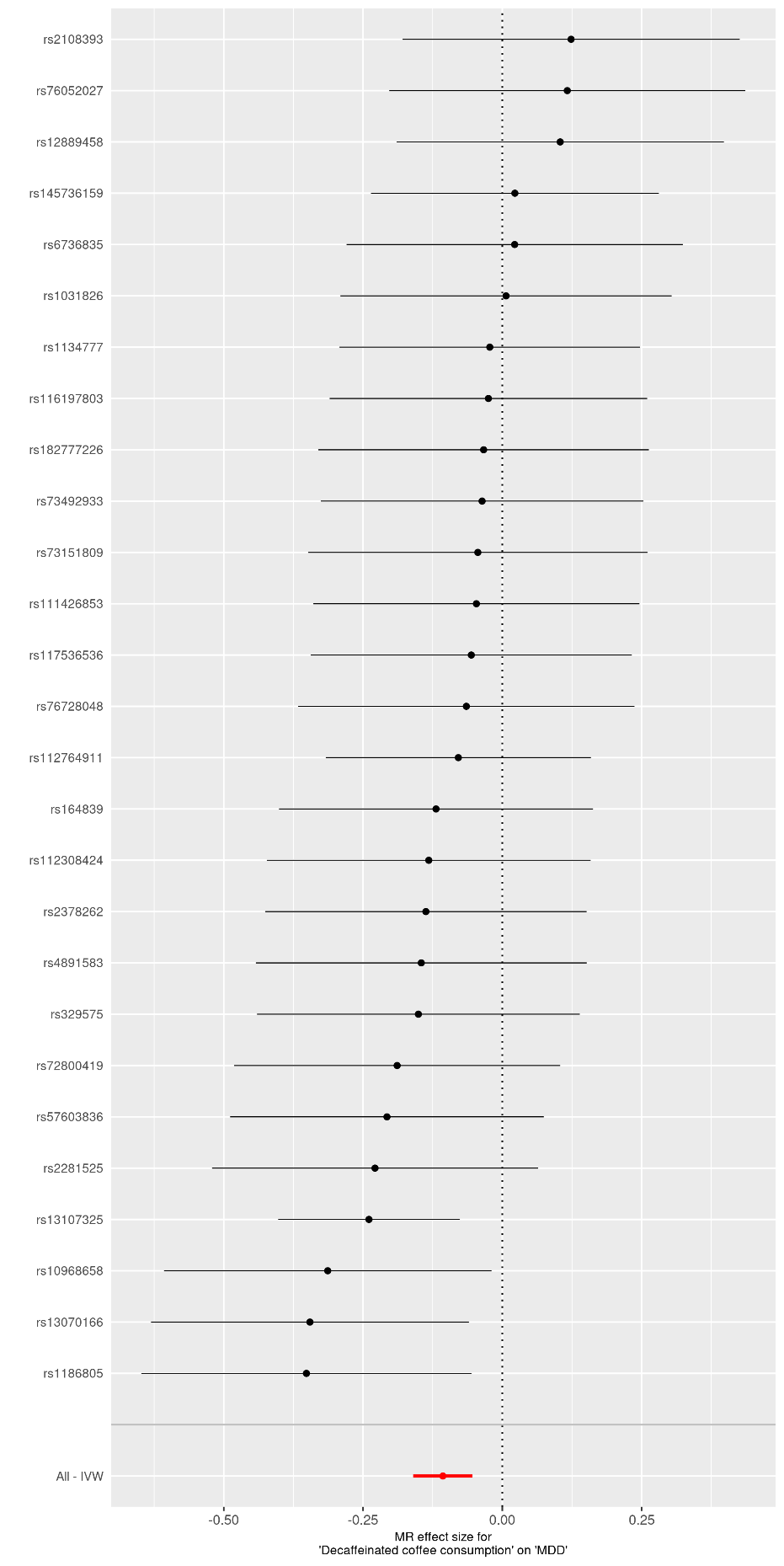


**Supplementary Figure 26.** Forest plot of MR analysis of AUDIT_T upon decaffeinated coffee consumption


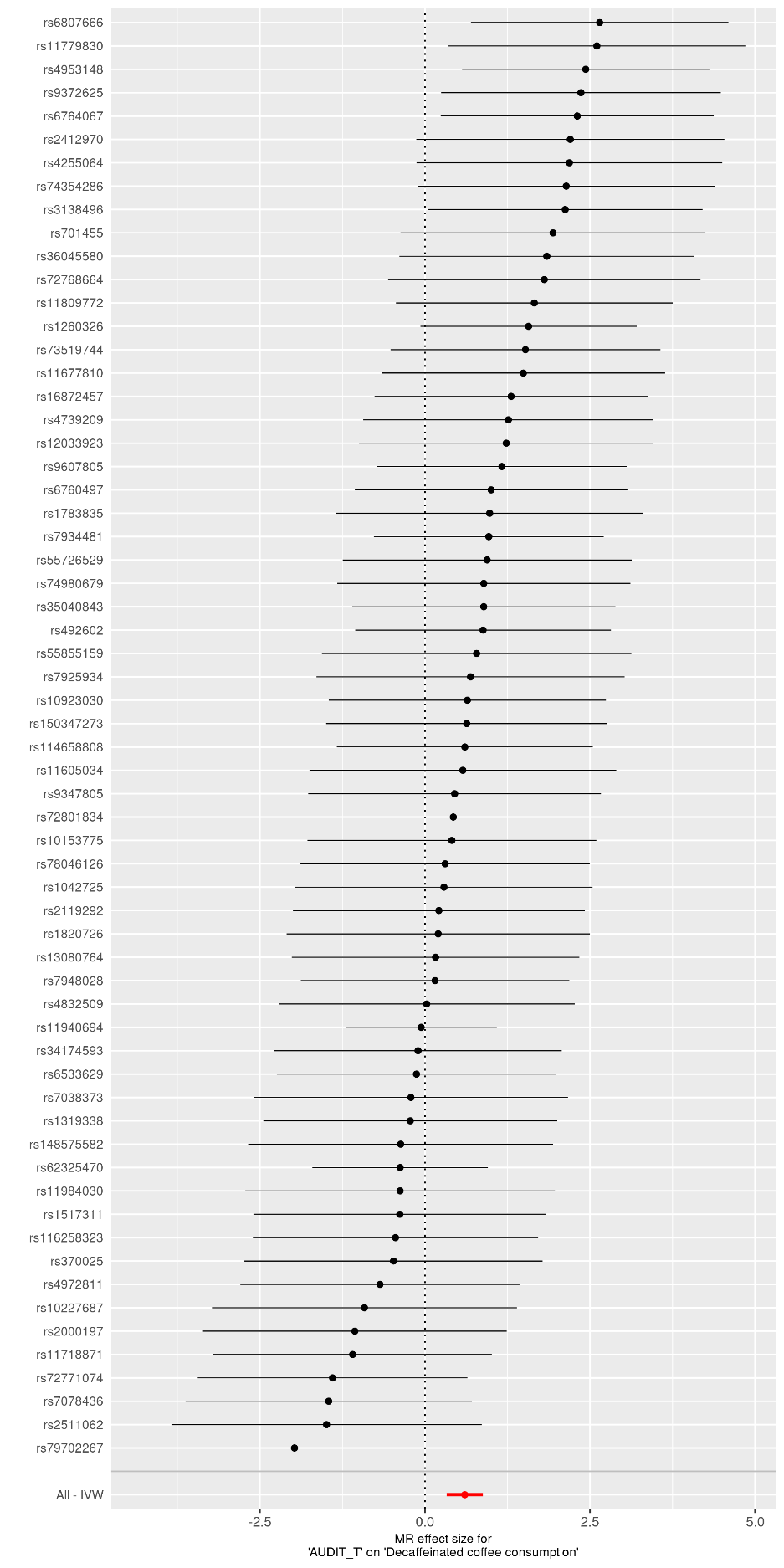


**Supplementary Figure 27.** Forest plot of MR analysis of AUDIT_C upon decaffeinated coffee consumption


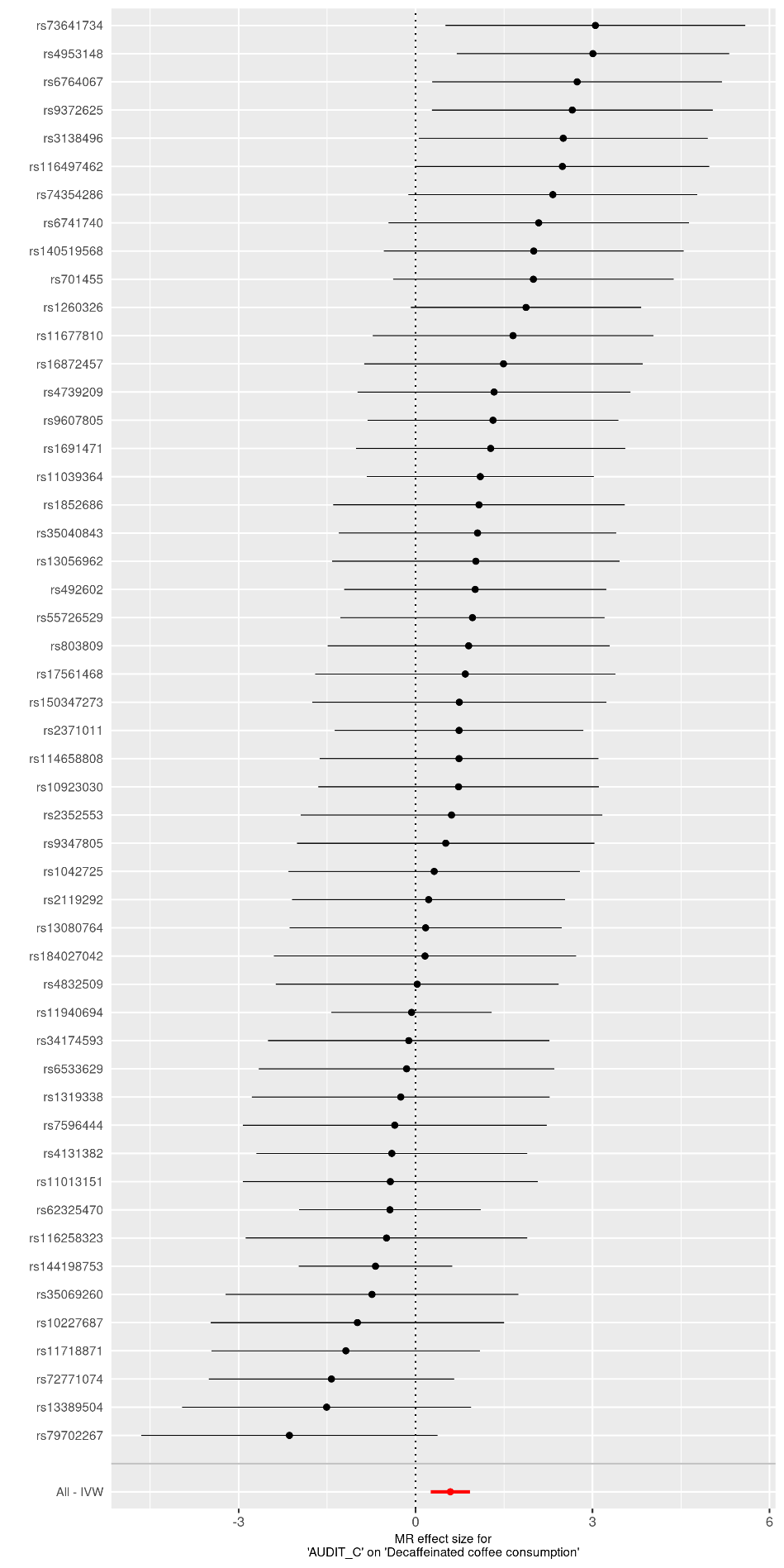


**Supplementary Figure 28.** Forest plot of MR analysis of Insomnia upon decaffeinated coffee consumption


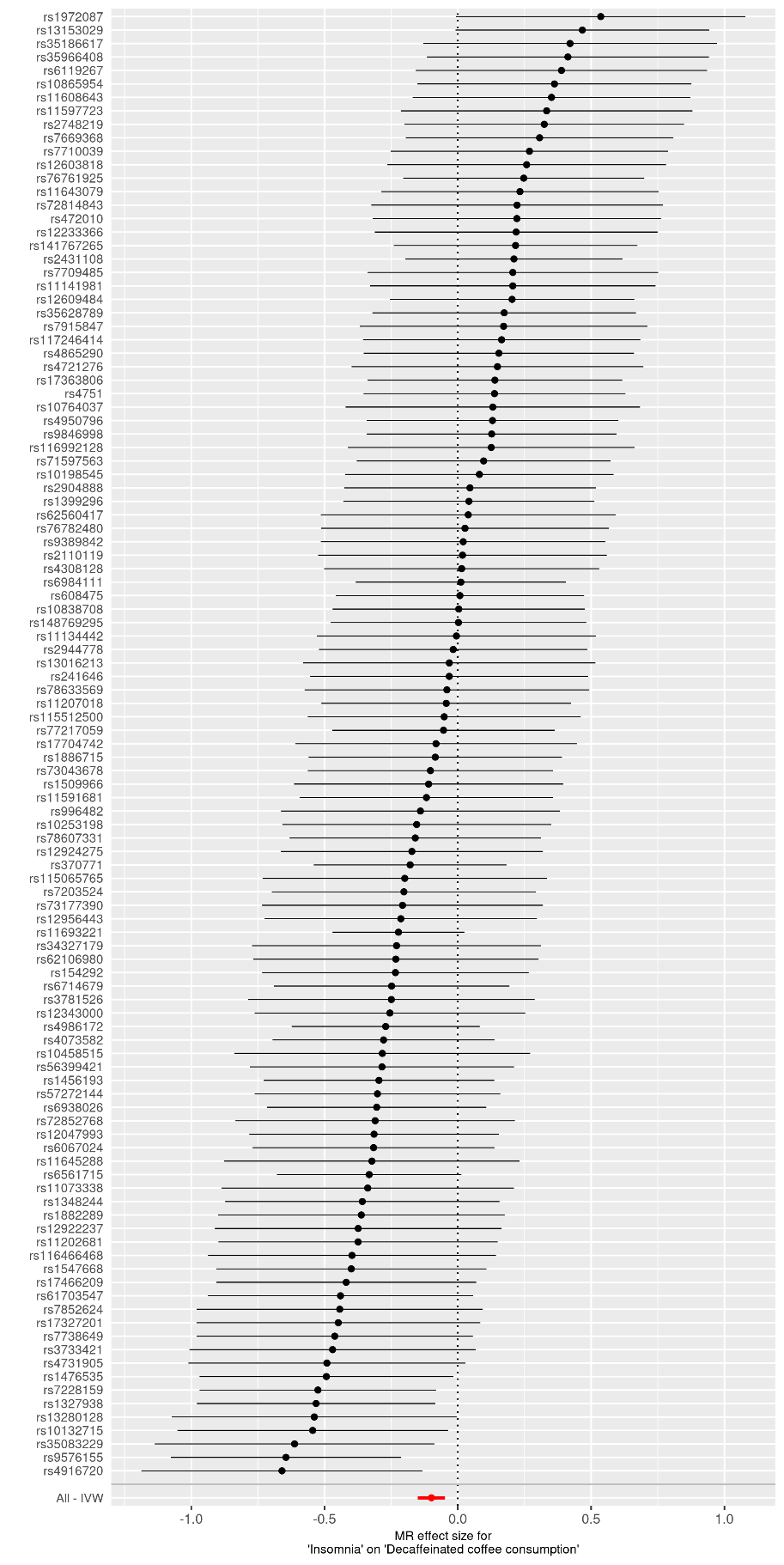


**Supplementary Figure 29.** Forest plot of MR analysis of MDD upon decaffeinated coffee consumption


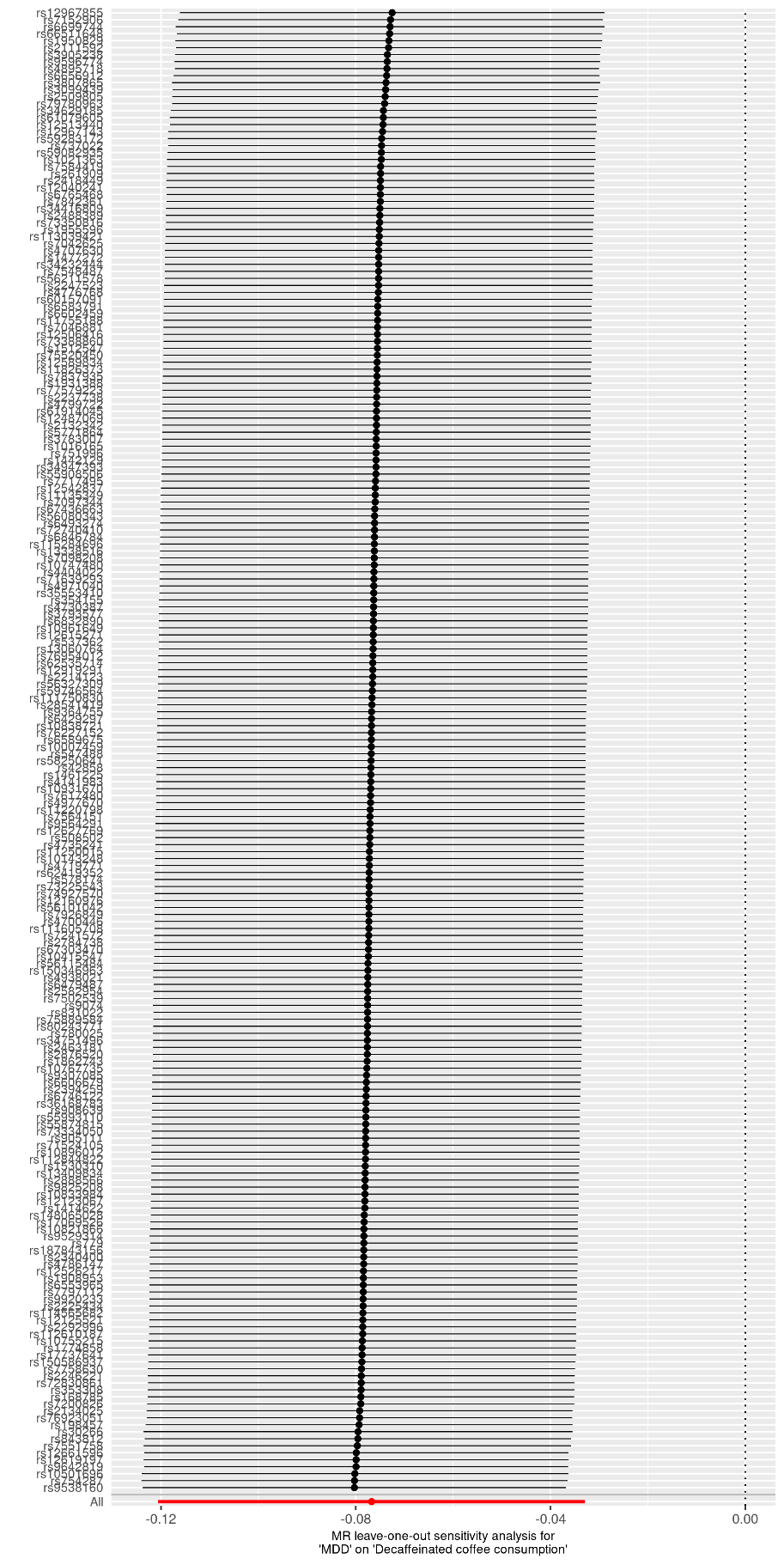


## Supplementary Tables

**
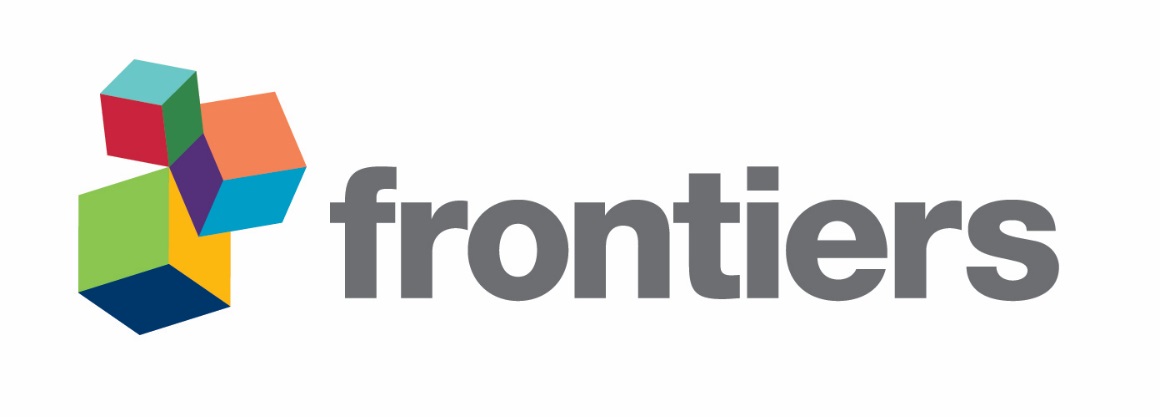
**

**Supplementary Table 1.** Information on data sources

**Supplementary Table 2.** Summary of the 75 genomic loci associated with decaffeinated coffee consumption and neuropsychiatric traits in cross-trait meta-analysis

**Supplementary Table 3.** The bidirectional MR analysis of decaffeinated coffee consumption and neuropsychiatric traits

**Supplementary Table 4.** Biological process of the shared gene set between decaffeinated coffee consumption and neuropsychiatric traits in Gene Ontology (GO) terms (FDR<0.05)

**Supplementary Table 5.** Pathway analysis of the shared gene set between decaffeinated coffee consumption and neuropsychiatric traits in Reactome pathways (FDR<0.05)

**Supplementary Table 6.** List of credible set SNPs in each locus from fine mapping

**Supplementary Table 7.** Co-location analysis of both decaffeinated coffee consumption and neuropsychiatric traits

**Supplementary Table 8.** TWAS results of decaffeinated coffee consumption (FDR<0.05)

**Supplementary Table 9.** TWAS results of Insomnia (FDR<0.05)

**Supplementary Table 10.** TWAS results of Neuroticism (FDR<0.05)

**Supplementary Table 11.** TWAS results of LCU (FDR<0.05)

**Supplementary Table 12.** TWAS results of MDD (FDR<0.05)

**Supplementary Table 13.** TWAS results of AUDIT_T (FDR<0.05)

**Supplementary Table 14.** TWAS results of AUDIT_C (FDR<0.05)

**Supplementary Table 15.** TWAS results of AUDIT_P (FDR<0.05)

**Supplementary Table 16.** Specific characteristics of genetic instructions of MR analysis of decaffeinated coffee consumption upon AD

**Supplementary Table 17.** Specific characteristics of genetic instructions of MR analysis of decaffeinated coffee consumption upon ALS

**Supplementary Table 18.** Specific characteristics of genetic instructions of MR analysis of decaffeinated coffee consumption upon ADHD

**Supplementary Table 19.** Specific characteristics of genetic instructions of MR analysis of decaffeinated coffee consumption upon BIP

**Supplementary Table 20.** Specific characteristics of genetic instructions of MR analysis of decaffeinated coffee consumption upon AN

**Supplementary Table 21.** Specific characteristics of genetic instructions of MR analysis of decaffeinated coffee consumption upon AUDIT_T

**Supplementary Table 22.** Specific characteristics of genetic instructions of MR analysis of decaffeinated coffee consumption upon AUDIT_C

**Supplementary Table 23.** Specific characteristics of genetic instructions of MR analysis of decaffeinated coffee consumption upon AUDIT_P

**Supplementary Table 24.** Specific characteristics of genetic instructions of MR analysis of decaffeinated coffee consumption upon insomnia

**Supplementary Table 25.** Specific characteristics of genetic instructions of MR analysis of decaffeinated coffee consumption upon LCU

**Supplementary Table 26.** Specific characteristics of genetic instructions of MR analysis of decaffeinated coffee consumption upon MDD

**Supplementary Table 27.** Specific characteristics of genetic instructions of MR analysis of decaffeinated coffee consumption upon Neuroticism

**Supplementary Table 28.** Specific characteristics of genetic instructions of MR analysis of decaffeinated coffee consumption upon SCZ

**Supplementary Table 29.** Specific characteristics of genetic instructions of MR analysis of AD upon decaffeinated coffee consumption

**Supplementary Table 30.** Specific characteristics of genetic instructions of MR analysis of ALS upon decaffeinated coffee consumption

**Supplementary Table 31.** Specific characteristics of genetic instructions of MR analysis of ADHD upon decaffeinated coffee consumption

**Supplementary Table 32.** Specific characteristics of genetic instructions of MR analysis of BIP upon decaffeinated coffee consumption

**Supplementary Table 33.** Specific characteristics of genetic instructions of MR analysis of AN upon decaffeinated coffee consumption

**Supplementary Table 34.** Specific characteristics of genetic instructions of MR analysis of AUDIT_T upon decaffeinated coffee consumption

**Supplementary Table 35.** Specific characteristics of genetic instructions of MR analysis of AUDIT_C upon decaffeinated coffee consumption

**Supplementary Table 36.** Specific characteristics of genetic instructions of MR analysis of AUDIT_P upon decaffeinated coffee consumption

**Supplementary Table 37.** Specific characteristics of genetic instructions of MR analysis of insomnia upon decaffeinated coffee consumption

**Supplementary Table 38.** Specific characteristics of genetic instructions of MR analysis of LCU upon decaffeinated coffee consumption

**Supplementary Table 39.** Specific characteristics of genetic instructions of MR analysis of MDD upon decaffeinated coffee consumption

**Supplementary Table 40.** Specific characteristics of genetic instructions of MR analysis of Neuroticism upon decaffeinated coffee consumption

**Supplementary Table 41.** Specific characteristics of genetic instructions of MR analysis of SCZ upon decaffeinated coffee consumption
